# Inflammation-triggered self-immolative conjugates enable oral peptide delivery by overcoming gastrointestinal barriers

**DOI:** 10.1126/sciadv.aea2989

**Published:** 2026-01-14

**Authors:** Juan Cheng, Peng Wu, Chenwen Li, Ying Han, Menglong Sun, Yin Dou, Sheng Chen, Jianxiang Zhang

**Affiliations:** ^1^ Department of Obstetrics and Gynecology, Chongqing Key Laboratory of Maternal and Fetal Medicine, The First Affiliated Hospital of Chongqing Medical University, Chongqing 400016, China.; ^2^ Department of Pharmaceutics, College of Pharmacy, Third Military Medical University (Army Medical University), Chongqing 400038, China.; ^3^ College of Pharmacy, Hanzhong Vocational and Technical College, Hanzhong, Shaanxi 723000, China.; ^4^ Yu-Yue Pathology Scientific Research Center, 313 Gaoteng Avenue, Jiulongpo District, Chongqing 400039, China.; ^5^ State Key Laboratory of Trauma and Chemical Poisoning, Third Military Medical University (Army Medical University), Chongqing 400038, China.; ^6^ Department of Pediatrics, Southwest Hospital, Third Military Medical University (Army Medical University), Chongqing 400038, China.

## Abstract

Oral delivery of peptide therapeutics remains challenging due to gastrointestinal (GI) degradation and poor intestinal absorption. Here, we propose a self-immolative peptide prodrug conjugate (SIPPC) platform for inflammation-targeted oral delivery, integrating a hydrophilic polyethylene glycol segment, a reactive oxygen species (ROS)–responsive hydrophobic self-immolative module, and a hydrolyzable scaffold, which collectively enable spontaneous assembly into micelle-like nanoparticles. Using three anti-inflammatory peptides (KPV, Ac-QAW, and IRW), we demonstrated that the engineered conjugates exhibit remarkable GI stability, efficient mucus penetration, and ROS-responsive release at inflamed sites. In colitis mice, the KPV-based conjugate (proKPV) achieved a 3.8-fold greater colonic accumulation than free KPV, with enhanced efficacy even at a 20-fold lower dose. Beyond therapeutic effects in the colitis model, oral proKPV substantially accumulated in inflamed lungs and exhibited potent anti-inflammatory efficacy in mice with acute lung injury. Ac-QAW and IRW-based conjugates exhibited comparable benefits, underscoring SIPPC as a transformative paradigm for oral peptide therapeutics, offering substantial promise for clinical translation in inflammatory disorders.

## Teaser

An inflammation-responsive, self-immolative prodrug conjugate strategy is developed for oral delivery of peptide therapeutics.

## INTRODUCTION

Peptide therapeutics have garnered remarkable attention owing to their high specificity, potent biological activity, and favorable toxicity profiles relative to small-molecule drugs (*1*–*3*). Peptides can mimic natural proteins and interact with specific molecular targets, including enzymes, receptors, and other signaling biomacromolecules, making them promising candidates for metabolic disorders, cancers, infections, and inflammatory conditions (*4*–*6*). The global peptide drug market is rapidly expanding, with over 100 approved agents and more than 300 candidates undergoing clinical trials (*7*). Semaglutide, a glucagon-like peptide-1 (GLP-1) receptor agonist, epitomizes this success, achieving ~$29 billion sales in 2024. Despite this potential, clinical translation remains challenges by oral delivery barriers, involving gastric acidity, enzymatic degradation (e.g., trypsin and carboxypeptidase), the gastrointestinal (GI) mucus layer, and the intestinal epithelial impermeability (*8*, *9*).

To protect peptides from gastric degradation, enteric coatings using pH-responsive polymers have been used to resist dissolution in acidic gastric environments and enable targeted intestinal release at higher pH. Furthermore, complementary citric acid incorporation induces transient luminal acidification, inhibiting peptidases to enhance enzymatic stability. Beyond pH adjustment, enzyme inhibitors, such as cholic acids and soybean trypsin inhibitor, are used to mitigate GI enzymatic degradation (*10*). For penetration enhancement, therapeutic peptides are formulated with mucolytic agents or mucus-penetrating enhancers, such as polyethylene glycol (PEG)–functionalized systems (*11*, *12*), zwitterionic polymer–coated nanoparticles (NPs) (*13*), and self-emulsifying drug delivery systems (*14*), mechanistically by reducing steric hindrance and modulating surface hydration. Biomimetic virus-inspired NPs further augment penetration efficiency, substantially improving oral bioavailability while preserving mucosal integrity (*15*). For the critical final step of intestinal epithelial translocation, permeation enhancers facilitate epithelial transport via transcellular or paracellular pathways (*16*–*18*). This approach is clinically validated by sodium *N*-(8-(2-hydroxybenzoyl)amino)caprylate (SNAC) in the oral semaglutide formulation Rybelsus (*19*). Furthermore, advances in intelligent manufacturing have catalyzed the development of ingestible multifunctional devices for spatiotemporally controlled drug delivery, encompassing self-propelled micro/nanorobots, GI microgrippers/microneedles, autoinjectable capsules, and self-orienting millimeter applicators (*20*–*25*). Despite these technological advancements, only a few peptide drugs, such as semaglutide and octreotide, have gained US Food and Drug Administration (FDA) approval in the past 5 years (*26*). Consequently, exploration of innovative systems capable of enhancing peptide stability, absorption efficiency, and targeted delivery of peptides has become imperative.

Self-immolative systems represent emerging platforms for precise drug delivery, using stimulus-triggered cascade degradation to achieve spatiotemporal control of drug release. These systems respond to endogenous stimuli [such as pH, glutathione, reactive oxygen species (ROS), and enzymes] or exogenous triggers (e.g., light and temperature), initiating intramolecular reactions in self-immolative linkers that drive sequential system breakdown and amplified payload release while preventing premature leakage (*27*, *28*). ROS-responsive systems are particularly advantageous due to their pathologically selective activation in disease microenvironments. Linkers such as boronate esters and thioketals maintain physiological stability yet undergo rapid cleavage in ROS-rich milieus (*29*–*31*). However, current platforms predominantly deliver small-molecule therapeutics (e.g., doxorubicin and gemcitabine) or small-molecule/gasotransmitter combinations (*32*, *33*), with peptide delivery systems remaining underdeveloped. In addition, most formulations are optimized for parenteral administration, whereas existing oral attempts fail to overcome intestinal mucus barriers, resulting in limited bioavailability. Compared to polymer-peptide conjugates, self-immolative systems exhibit superior safety through complete activation-triggered hydrolysis into small-molecule metabolites with efficient renal/hepatic clearance (*34*, *35*).

**Fig. 1.**
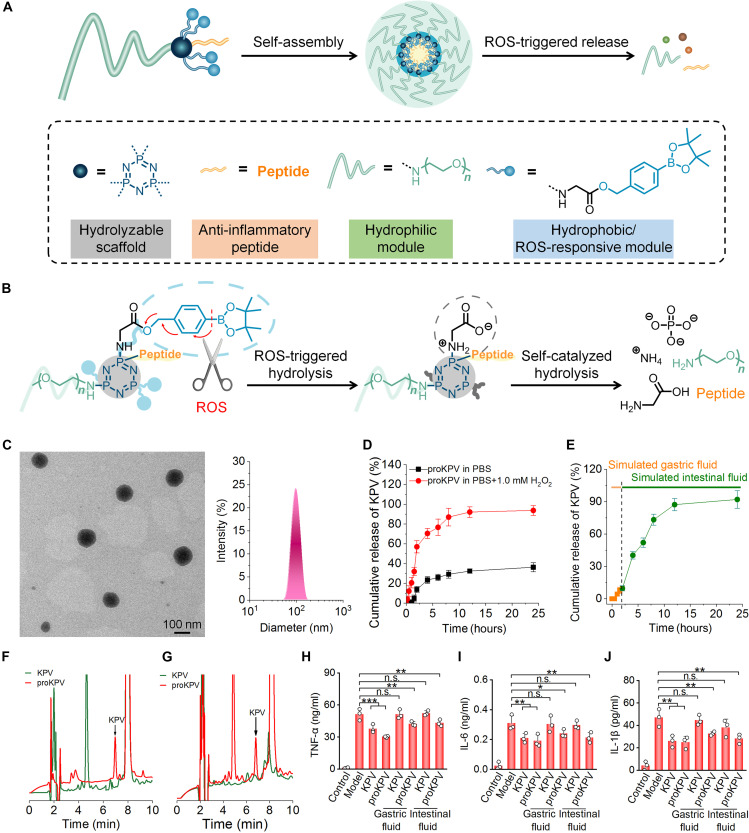
Design strategies for inflammation-responsive, self-immolative peptide prodrugs and characterization of a peptide KPV prodrug conjugate (proKPV). (**A**) Schematic of engineered inflammation-triggered self-immolative amphiphilic conjugates as prodrug delivery platforms for peptides. The peptide is conjugated to a hydrolyzable scaffold molecule HCCP, with a simultaneously attached PEG chain to enhance hydrophilicity and stability. In addition, an inflammation-responsive hydrophobic unit (PBE) is covalently linked to HCCP via a glycine residue, imparting amphiphilicity and a triggerable self-immolative property to the final conjugate. The engineered peptide conjugates can self-assemble into micelle-like NPs, encapsulating the peptide segments within the core. (**B**) Hydrolytic mechanism of the engineered inflammation-responsive self-immolative peptide conjugate. Under inflammatory and oxidative conditions, ROS trigger hydrolysis of the PBE unit, exposing the glycine moiety, which subsequently induces self-immolative cleavage of the HCCP scaffold, releasing the pristine peptide. (**C**) TEM image (left) and size distribution profile (right) of NPs assembled by proKPV. (**D** and **E**) In vitro release profiles of KPV from proKPV NPs in 0.01 M PBS at pH 7.4 with or without 1.0 mM H_2_O_2_ (D), as well as in solutions simulating GI conditions (E). (**F** and **G**) Representative HPLC curves indicate chemical stability of free KPV or proKPV after incubation in simulated gastric (F) or intestinal (G) fluids. (**H** to **J**) Levels of TNF-α (H), IL-6 (I), and IL-1β (J) secreted by macrophages subjected to different treatments. RAW264.7 macrophages were stimulated with LPS and subsequently incubated with either KPV or proKPV (containing equivalent concentrations of KPV), with or without pretreatment in simulated gastric and intestinal fluids. Cells in the control and model groups were treated with fresh medium without or with LPS, respectively. Data in (D), (E), and (H) to (J) are presented as means ± SD (*n* = 3). *P < 0.05; **P < 0.01; ***P < 0.001; n.s., not significant.

Herein, we present a self-immolative peptide prodrug conjugate (SIPPC) strategy for inflammation-targeted peptide delivery, specifically designed for oral administration to inflamed tissues. The rationally engineered conjugate comprises four key components: a hydrophilic chain, an inflammation-responsive hydrophobic self-immolative moiety, a bioactive peptide payload, and a hydrolyzable scaffold (Fig. 1A). Its amphiphilic nature enables self-assembly into micelle-like NPs that encapsulate peptides within hydrophobic cores, shielding them from harsh GI environments following oral delivery. Critically, surface-exposed hydrophilic chains facilitate dual penetration through mucus and epithelial barriers. To validate this approach, we used three candidate anti-inflammatory peptides. Ulcerative colitis, a representative inflammatory bowel disease (IBD), and acute lung injury (ALI), a typical inflammatory pulmonary disorder, were used as disease models. Notably, both colitis and ALI are characterized by local oxidative stress, a key pathological feature underlying the progression of these inflammatory disorders (*36*–*38*). Pathological ROS overproduction in the diseased tissue provides an implementation window for orally administered, ROS-responsive self-immolative systems to deliver peptide therapeutics. At inflamed sites, ROS trigger hydrolysis of the hydrophobic unit, exposing a carboxyl group that initiates scaffold self-immolation. This cascade culminates in precisely controlled release of pristine therapeutic peptides (Fig. 1B), yielding superior efficacy versus free peptides and a clinically used positive control.

## RESULTS

### Design, synthesis, and characterization of an inflammation-responsive self-immolative peptide prodrug proKPV

We propose an inflammation-responsive SIPPC strategy for targeted oral peptide delivery to inflammatory sites (Fig. 1A). Specifically, this design involves conjugation of therapeutic peptides to a hydrolyzable hexachlorocyclotriphosphazene (HCCP) scaffold, modified with PEG chains to improve hydrophilicity and stability. A ROS-responsive hydrophobic unit, i.e., 4-(hydroxymethyl)phenylboronic acid pinacol ester (PBE), is further incorporated via glycine linkage, endowing the conjugate with both amphiphilicity and ROS-triggered self-immolation capability. This engineered prodrug can spontaneously form micelle-like NPs that package peptide segments within their cores, providing protection against GI degradation while enabling inflammation-targeted delivery. Upon reaching inflamed tissues, elevated ROS levels mediate PBE hydrolysis, exposing the glycine moiety and initiating sequential self-immolation of the HCCP scaffold, ultimately releasing native therapeutic peptides.

As a proof of concept, we initially used the tripeptide KPV (Lys-Pro-Val) as a model anti-inflammatory peptide. Derived from α-melanocyte–stimulating hormone, KPV inhibits tumor necrosis factor–α (TNF-α) signaling to suppress inflammation (*39*). An inflammation-responsive amphiphilic KPV prodrug (proKPV) was synthesized via a facile nucleophilic reaction of HCCP with amine-terminated PEG [PEG-NH_2_; weight-average molecular weight (*M_w_*) = 2 kDa], glycine-conjugated PBE (Gly-PBE), and KPV (figs. S1 to S3). Structural characterization by ^1^H nuclear magnetic resonance (NMR) and Fourier transform infrared (FTIR) spectroscopy confirmed successful formation of proKPV (fig. S4), with one PEG chain, four PBE units, and one peptide moiety in each proKPV. Notably, proKPV exhibited excellent solubility in aqueous solutions and common organic solvents such as acetonitrile, methanol, and ethanol.

Subsequently, we investigated ROS-responsive hydrolysis profiles of proKPV. After incubation with 10 mM hydrogen peroxide (H_2_O_2_), a major component of ROS, measurements by ^1^H NMR, ^31^P NMR, and electrospray ionization (ESI) mass spectrometry confirmed complete hydrolysis of proKPV into renally excretable and water-soluble molecules, including pinacol, *p*-(hydroxymethyl)phenol (HMP), glycine, PO_4_^3−^, PEG-NH_2_, and free KPV (fig. S5, A to C). High-performance liquid chromatography (HPLC) analysis further revealed the H_2_O_2_-triggered release of KPV from the prodrug proKPV (fig. S5D). Accordingly, we propose the hydrolysis mechanism of proKPV (fig. S5E). In the presence of H_2_O_2_, PBE undergoes initial oxidation, generating *p*-quinone methide and pinacol boronate ester intermediates that further decompose into pinacol, boric acid, and HMP. Concurrently, the resultant Gly-substituted phosphazene can be rapidly hydrolyzed into glycine, ammonium, and PO_4_^3−^ through carboxyl-catalyzed self-immolative ring opening while simultaneously liberating PEG and KPV by phosphazene ring cleavage.

### Self-assembly, in vitro release, and biophysicochemical stability of proKPV under different conditions

Given the amphiphilic nature of proKPV, we then examined its self-assembly behaviors in aqueous solutions. Transmission electron microscopy (TEM) observation indicated that proKPV formed nearly spherical NPs (Fig. 1C, left). Measurement by dynamic light scattering (DLS) showed a narrow size distribution of proKPV NPs (Fig. 1C, right), with an average diameter of 81.0 ± 1.0 nm and ζ-potential of 2.9 ± 0.1 mV. proKPV NPs exhibited excellent stability upon incubation in water, saline, phosphate-buffered saline (PBS), and fetal bovine serum (FBS) (fig. S6). Using the pyrene-based fluorescence probe method (*40*), we determined the critical micelle concentration (CMC) of proKPV to be ~49 μg/ml (fig. S7). Consequently, proKPV can self-assemble into well-defined NPs in aqueous solutions. Then, in vitro release kinetics of KPV from proKPV NPs were evaluated in PBS at pH 7.4 with or without 1.0 mM H_2_O_2_. proKPV NPs showed rapid release in the presence of H_2_O_2_, with nearly complete KPV release occurring within 24 hours. By contrast, the release profile was significantly slower in H_2_O_2_-free PBS (Fig. 1D). Further investigation of KPV release under simulated GI conditions revealed that only a minor fraction (~9.0%) of KPV was released after 2 hours of incubation in simulated gastric fluid (SGF). However, upon transition to simulated intestinal fluid (SIF) containing 1.0 mM H_2_O_2_, the release rate of KPV remarkably increased (Fig. 1E). These findings are consistent with our previous observation that the PBE moiety remains stable in acidic environments, even in the presence of H_2_O_2_ (*41*). Collectively, our results demonstrate that proKPV NPs can effectively protect KPV from premature release under gastric conditions while enabling ROS-responsive and intestine-specific drug release.

Subsequently, the protective capacity of proKPV-assembled NPs against GI degradation was evaluated by exposing both free KPV and proKPV NPs to SGF and SIF. Following 2-hour incubation, HPLC analysis indicated nearly complete hydrolysis of free KPV in both media (Fig. 1, F and G), whereas proKPV NPs effective preserved KPV integrity under identical conditions. In addition, proKPV maintained structural integrity following 2-hour incubation in pH 2 hydrochloric acid, as evidenced by negligible variation in the diameter, polydispersity index (PDI), and molecular weight (figs. S8 and S9). We next examined the anti-inflammatory activity of proKPV following GI exposure. In lipopolysaccharide (LPS)–stimulated RAW264.7 mouse macrophages, proKPV significantly inhibited the expression of key inflammatory cytokines, such as TNF-α, interleukin-1β (IL-1β), and IL-6, regardless of prior incubation with SIF or SGF. Notably, the anti-inflammatory potency of preincubated proKPV was comparable to that of untreated proKPV. By contrast, free KPV subjected to GI conditions completely lost its ability to inhibit pro-inflammatory cytokine production in LPS-induced macrophages (Fig. 1, H to J), agreeing with its instability under such conditions. These findings conclusively demonstrate that our developed proKPV formulation not only enhances KPV stability in harsh GI environments but also fully maintains its therapeutic bioactivity.

### In vitro biological effects of proKPV in inflammatory cells

Local inflammatory responses and oxidative stress–mediated tissue damage critically contribute to the pathogenesis of inflammatory diseases (*42*–*44*). In particular, neutrophils and macrophages act as key mediators, driving disease progression by epithelial barrier disruption, oxidative and proteolytic tissue damage, and persistent secretion of pro-inflammatory cytokines/chemokines (*45*, *46*). Given the pivotal role of these cells in inflammation, we evaluated biological activities of proKPV in both neutrophils and macrophages.

**Fig. 2.**
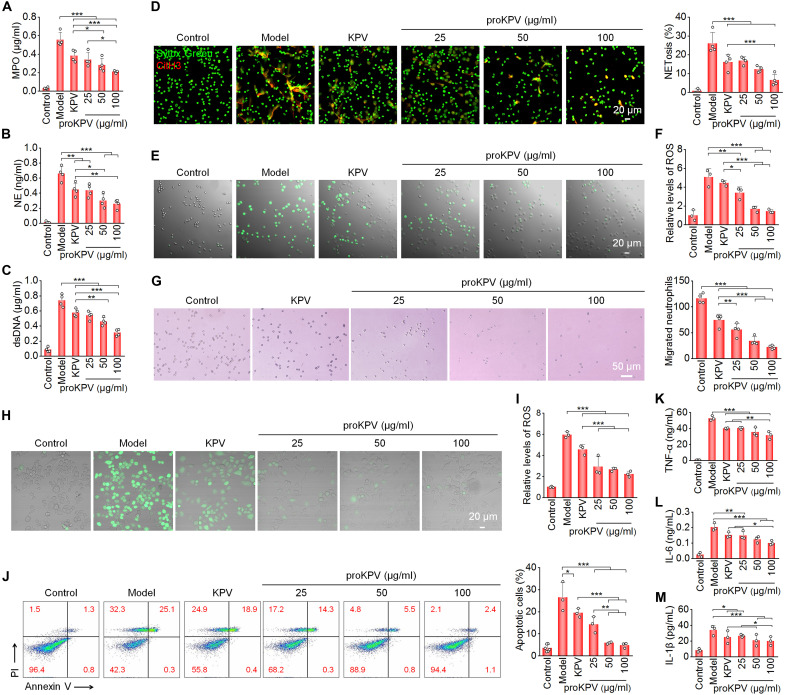
Evaluations of in vitro activities of proKPV. (**A** to **C**) Levels of MPO (A), NE (B), and dsDNA (C) expressed by neutrophils after stimulation with PMA and treatment with different formulations. (**D**) Representative confocal microscopy images (left) and quantitative analyses (right) of NET generation after neutrophils were stimulated with PMA and treated with different formulations. (**E** and **F**) Confocal microscopy images showing intracellular ROS generation (E) and flow cytometric quantification of relative ROS levels (F) in neutrophils. (**G**) Optical microscopic images (left) and quantitative analysis (right) showing migrated neutrophils after different treatments. (**H**) Fluorescence microscopy images showing intracellular ROS generation in macrophages stimulated with PMA and treated with different formulations for 2 hours. DCFH-DA was used as a fluorescent probe to stain intracellular ROS. (**I**) Relative ROS levels in macrophages quantified by flow cytometry. (**J**) Flow cytometry plots (left) and corresponding quantitative analysis (right) indicate ROS-induced apoptosis of macrophages following different treatments. Before exposure to H_2_O_2_ for 12 hours, macrophages were preincubated with different formulations for 4 hours. (**K** to **M**) Expression levels of TNF-α (K), IL-6 (L), and IL-1β (M) in macrophages after different treatments. Macrophages were preincubated with different formulations and LPS for 6 hours before cytokine analysis. In all cases, cells in the KPV group were treated with KPV (10 μg/ml), whereas cells in different proKPV groups were treated with proKPV (25, 50, or 100 μg/ml). Data are presented as means ± SD (*n* = 3 to 4 biological replicates). *P < 0.05; **P < 0.01; ***P < 0.001.

The biological effects of proKPV were first assessed in neutrophils. To examine cellular uptake, a cyanine5 (Cy5)–labeled fluorescent conjugate (PCy5) was synthesized and confirmed by ^1^H NMR spectroscopy (fig. S10). Cy5-labeled proKPV NPs (Cy5-proKPV NPs) were prepared by coassembly of proKPV with PCy5 at a 10:1 weight ratio. Both confocal microscopy observation and flow cytometric quantification revealed dose-dependent and time-dependent internalization of Cy5-proKPV NPs in neutrophils (figs. S11 and S12). Increasing evidence implicates critical roles of neutrophil extracellular traps (NETs) in the pathogenesis of IBD and other inflammatory disorders, where they contribute to intestinal barrier dysfunction, homeostasis disruption, and extracellular matrix (ECM) degradation (*47*). Elevated levels of NETs have been consistently detected in patients with IBD and animal models of colitis (*48*, *49*). Consequently, the effects of proKPV treatment on NET formation were evaluated. In phorbol 12-myristate 13-acetate (PMA)–stimulated neutrophils, we observed significant up-regulation of key NET components including myeloperoxidase (MPO), neutrophil elastase (NE), and extracellular double-stranded DNA (dsDNA), all of which were dose-dependently suppressed by proKPV treatment (Fig. 2, A to C). Immunofluorescence analysis further indicated that proKPV at varying doses substantially reduced both the citrullinated histone H3 (CitH3) area and the extent of NETosis in activated neutrophils (Fig. 2D). Consistent with these findings, proKPV significantly reduced PMA-induced ROS generation in a dose-responsive manner (Fig. 2, E and F). In addition, transwell migration assay showed that proKPV dose-dependently inhibited neutrophil migration (Fig. 2G). Accordingly, proKPV can effectively suppress neutrophil activation and NETosis. In all these cases, proKPV showed enhanced therapeutic potency relative to the same dose of free KPV.

We next investigated antioxidative and anti-inflammatory properties of proKPV in RAW264.7 macrophages. Confocal microscopy and flow cytometry analyses revealed that proKPV treatment at various doses effectively suppressed PMA-induced intracellular ROS generation (Fig. 2, H and I). The superior antioxidative capacity of proKPV was further demonstrated by its dose-dependent protection against ROS-mediated cellular apoptosis (Fig. 2J). In LPS-activated macrophages, proKPV also showed enhanced anti-inflammatory effects, significantly reducing the elevated levels of TNF-α, IL-6, and IL-1β more potently than free KPV treatment (Fig. 2, K to M). These results collectively demonstrate that the amphiphilic prodrug formulation proKPV substantially enhances biological activities of KPV. The improved pharmacological profile of proKPV encompasses multiple beneficial effects, such as potent antioxidant activity, inhibition of neutrophil infiltration, suppression of NETosis, protection against ROS-induced apoptosis, and significant reduction of pro-inflammatory cytokine secretion.

### Colonic permeability of orally delivered proKPV NPs in colitic mice

**Fig. 3.**
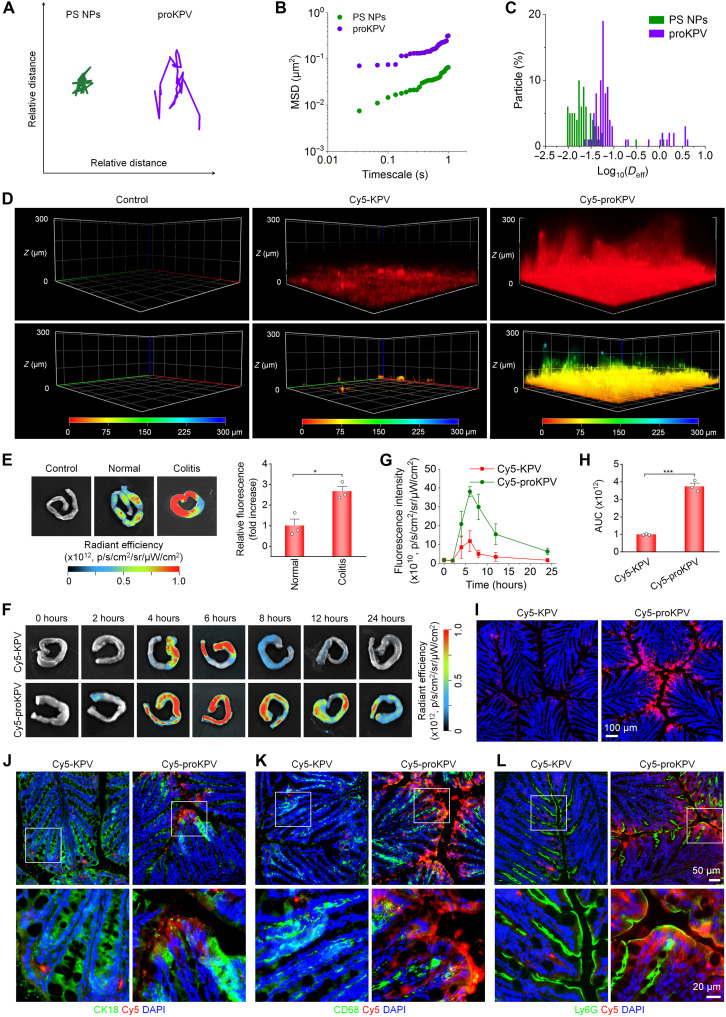
Permeability ability and selective accumulation of proKPV in the inflamed colons of mice with acute colitis. (**A**) Representative trajectories of Cy5-proKPV NPs and Cy5-labeled PS NPs in the mouse intestinal mucus, as determined by MTrackJ analysis of 5-min confocal microscopy recordings. (**B**) Time-dependent ensemble-averaged geometric MSD of proKPV NPs and PS NPs in the mouse intestinal mucus. (**C**) Distributions of log(*D*_eff_) for proKPV NPs and PS NPs at a 5-min timescale, with at least 100 particles tracked per sample for each type. (**D**) Three-dimensional fluorescence images illustrate the penetration of Cy5-KPV and Cy5-proKPV NPs through the mouse intestinal mucus. (**E**) Representative ex vivo images (left) and quantitative analysis (right) illustrating accumulation of Cy5-labeled proKPV in the colons of mice with or without DSS-induced acute colitis at 6 hours after oral administration. (**F** and **G**) Fluorescence images (F) and quantitative analysis (G) of the colonic tissues isolated from colitis mice at different time points following treatment with free Cy5-KPV or Cy5-proKPV. (**H**) AUC of Cy5 fluorescence in colonic tissues after treatment with free Cy5-KPV or Cy5-proKPV. (**I**) Fluorescence images of cryosections of colonic tissues from colitis mice at 6 hours after treatment with Cy5-KPV or Cy5-proKPV. Nuclei were stained with DAPI (blue). (**J** to **L**) Immunofluorescence analysis of colocalization of Cy5-KPV or Cy5-proKPV with CK18^+^ intestinal epithelial cells (J), CD68^+^ macrophages (K), or Ly6G^+^ neutrophils (L) in cryosections of colonic tissues. Data in (E), (G), and (H) are presented as means ± SD (*n* = 3 biological replicates). *P < 0.05; ***P < 0.001.

Subsequently, we evaluated the mucus-penetrating capability of proKPV NPs using multiple particle tracking technology. Using polystyrene NPs (PS NPs) as a control, comparative analysis revealed substantially higher mobility of proKPV NPs in freshly isolated mucus, demonstrating faster diffusion rates and wider distribution within 5 min (Fig. 3A). Quantitative assessment of particle transport constraints by calculating timescale–dependent mean squared displacement (MSD) confirmed superior mobility of proKPV NPs across all measured timescales (Fig. 3B). Further characterization of individual particle movements showed that proKPV NPs exhibited notably higher effective diffusivity (*D*_eff_) values compared to PS NPs (Fig. 3C).

On the basis of these in vitro observations, we examined colonic penetration of proKPV NPs in vivo. Colitis was induced in mice by oral administration of 3% dextran sulfate sodium (DSS) in drinking water (*50*). Confocal microscopy *z*-stack analysis of colon sections from colitic mice at 6 hours postadministration revealed deep *z*-directional distribution of Cy5-proKPV NP fluorescence, whereas free Cy5-KPV remained confined to superficial layers (Fig. 3D). These results demonstrate that proKPV NPs exhibit exceptional capacity for mucus layer penetration and enhanced colonic permeability. The observed penetration enhancement may be ascribed to the small size of proKPV NPs and PEG-facilitated mucus diffusion. This combination reduces nanocarrier-mucus affinity by minimizing electrostatic/hydrophobic interactions, preventing protein corona formation, and inhibiting NP aggregation (*11*, *51*).

### Biodistribution and colon-targeting effects of orally delivered proKPV in colitis mice

Before therapeutic assessment of proKPV, we investigated the GI transport and inflammation-targeting capacity of orally administered Cy5-proKPV NPs in mice with DSS-induced colitis. At 6 hours postadministration, ex vivo imaging revealed significantly enhanced fluorescence signals in the colons of colitis mice compared to healthy controls (Fig. 3E, left), with the inflamed tissues exhibiting a 2.7-fold increase in the fluorescence intensity (Fig. 3E, right). This preferential accumulation in the inflamed colon likely results from epithelial barrier dysfunction, which substantially enhances particulate permeability in colitic regions (*36*). Meanwhile, Cy5-proKPV NPs displayed systemic translocation to major organs (such as the heart, liver, lung, and kidneys) in both healthy and colitis mice (fig. S13), with enhanced fluorescence intensities in the colitis group. Although colitis-induced epithelial barrier disruption promotes organ accumulation, the observed distribution in healthy controls provides direct evidence that proKPV NPs overcome physiological barriers and transport across the intact intestinal epithelium.

To further interrogate the intestinal retention and bioavailability of proKPV NPs, we performed a comparative evaluation of proKPV NPs versus free KPV in colitic mice. At 4 hours following oral delivery, mice treated with Cy5-proKPV NPs exhibited substantially higher fluorescence intensities in inflamed colons compared to those treated with Cy5-KPV. Notably, Cy5-proKPV NPs demonstrated sustained colonic retention with detectable fluorescence persisting for 24 hours (Fig. 3, F and G), whereas Cy5-KPV signals diminished substantially by 12 hours and became negligible by 24 hours. Pharmacokinetic analysis showed the area under the fluorescence intensity–time curve (AUC) of Cy5-proKPV NPs in the inflamed colon was 3.8-fold higher than free Cy5-KPV (Fig. 3H). Meanwhile, colitis mice administered with Cy5-proKPV exhibited relatively high fluorescence intensity in the blood, which persisted for up to 24 hours. In contrast, the free Cy5-KPV group displayed both lower fluorescence intensity and a shorter duration of fluorescence in the blood (fig. S14).

Fluorescence observation of colonic tissue cryosections confirmed enhanced targeting of Cy5-proKPV NPs to inflamed colonic microvilli, compared to Cy5-KPV (Fig. 3I). Furthermore, immunofluorescence analyses revealed substantial localization of Cy5-proKPV NPs in CK18^+^ intestinal epithelial cells, CD68^+^ macrophages, and Ly6G^+^ neutrophils (Fig. 3, J to L), with particularly high colocalization observed in macrophages and neutrophils. Together, these findings demonstrate that orally delivered proKPV NPs can achieve effective accumulation in inflamed colons, prolonged tissue retention, and targeted delivery to pathologically relevant epithelial cells and inflammatory cells. The superior colonic bioavailability of proKPV NPs compared to free KPV primarily stems from their enhanced GI stability. Oral proKPV exhibits notably higher systemic bioavailability than KPV, due to both improved stability and enhanced mucus penetration capacity.

### Therapeutic effects, safety assessment, and mechanistic evaluations of proKPV in mice with acute colitis

**Fig. 4.**
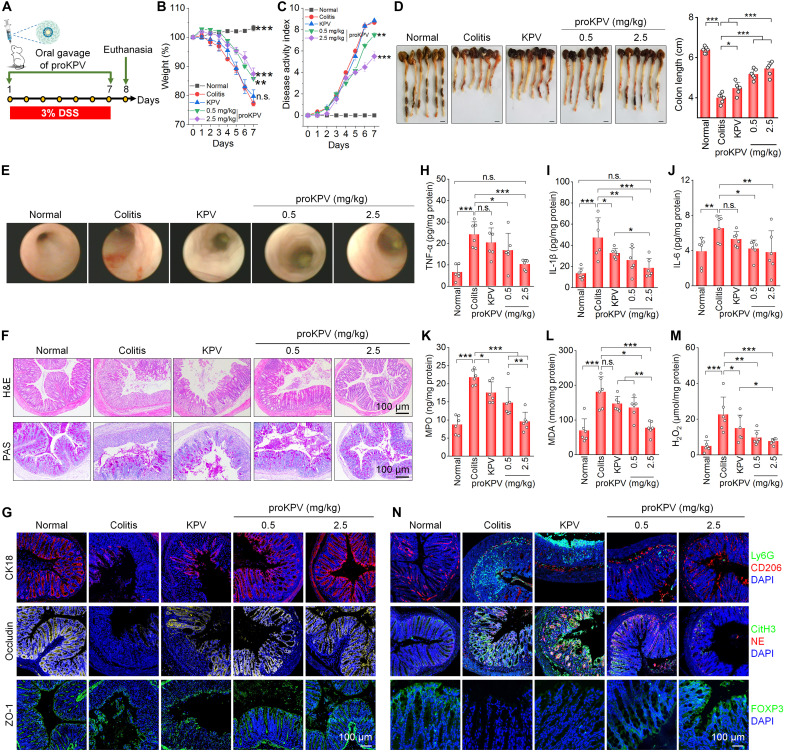
In vivo therapeutic effects of orally delivered proKPV in mice with DSS-induced acute colitis. (**A**) Schematic illustration of treatment regimens. (**B**) Body weight of mice during a 7-day treatment course. Data were normalized as the percentages of the body weight at day 0.**P < 0.01; ***P < 0.001; n.s., not significant versus the colitis group. (**C**) Changes in DAI. **P < 0.01 and ***P < 0.001 versus the colitis group. (**D**) Digital photos (left) and quantified lengths (right) of colonic tissues isolated from mice at day 7 following different treatments. Scale bars, 5 mm. (**E**) Representative miniendoscopic images of colons from mice after 7 days of treatment. (**F**) Colonic tissue sections stained with H&E or PAS for different groups. (**G**) Immunofluorescence analysis of the expression of CK18, occludin, and ZO-1 in colonic tissues from mice following various treatments. Scale bars, 100 μm. (**H** to **M**) Levels of TNF-α (H), IL-1β (I), IL-6 (J), MPO (K), MDA (L), and ROS (M) in colonic tissues isolated from healthy or diseased mice treated with different formulations. After 7 days of treatment, colonic tissue homogenates were prepared, and mediator concentrations were quantified and normalized to the total protein content. (**N**) Immunofluorescence of colonic tissues showing Ly6G^+^ neutrophils, CD206^+^ M2 macrophages, CitH3/NE-positive NETs, and FOXP3^+^ T_reg_ cells after different treatments. Data in (B) to (D) and (H) to (M) are presented as means ± SD (*n* = 6 biological replicates). *P < 0.05; **P < 0.01; ***P < 0.001; n.s., not significant.

The above findings substantiate that the amphiphilic prodrug of KPV substantially improves its chemical stability while enhancing both mucus-penetrating capability and colonic targeting efficiency. We subsequently performed in vivo studies to assess therapeutic efficacy of proKPV in mice with DSS-induced colitis, using KPV as a control (Fig. 4A). Oral administration of proKPV at 0.5 and 2.5 mg/kg for 7 consecutive days in DSS-challenged mice provided robust protection against DSS-induced body weight loss, elevated disease activity index (DAI), and colon shortening (Fig. 4, B to D). By contrast, free KPV at 1 mg/kg (equivalent to the peptide dose of 10 mg/kg proKPV) showed no beneficial effects. Miniendoscopy and histological analysis of the model group revealed severe ulcers, granular mucosal surface, extensive disruption of colonic epithelium, and massive inflammatory cell infiltration. Treatment with proKPV, particularly at 2.5 mg/kg, effectively ameliorated colitis, as evidenced by reduced inflammatory cell infiltration, nearly complete restoration of the colonic mucosa, and substantially normalized histological microstructure (Fig. 4, E and F). Periodic acid–Schiff (PAS) staining further confirmed that proKPV treatment substantially mitigated goblet cell loss and restored the mucus layer, closely resembling the normal group (Fig. 4F).

As previously established, epithelial barrier disruption represents a hallmark pathological feature of colitis (*52*, *53*). The structural integrity of this biological barrier is closely associated with the expression of the epithelial marker cytokeratin 18 (CK18) and key tight junction proteins such as occludin and zonula occludens-1 (ZO-1) (*54*, *55*), which serve as critical regulators of mucosal permeability (*56*). Immunofluorescence analysis indicated that oral treatment with proKPV markedly enhanced the expression of CK18, occludin, and ZO-1 across all tested doses, demonstrating effective restoration of intestinal barrier integrity (Fig. 4G). In contrast, both model and KPV groups showed minimal expression of these proteins, confirming their limited effects on epithelial function. CK18 is a well-recognized biomarker associated with the severity and treatment response in colitis. ZO-1 plays a pivotal role in nucleating junctional complex assembly and enhancing transepithelial resistance. Occludin seals the paracellular “leak pathway,” and its degradation increases macromolecular flux. Conversely, therapeutic restoration of occludin reduces intestinal permeability and inflammation. Together, the recovery of these tight junction proteins restricts luminal antigen penetration and attenuates inflammatory cascades (*56*, *57*). Supporting these findings, proKPV treatment substantially reduced systemic exposure to orally administered fluorescein isothiocyanate (FITC)–dextran in colitis mice, providing functional evidence of barrier repair (fig. S15).

Furthermore, proKPV therapy effectively alleviated colitis-associated splenomegaly and renomegaly (fig. S16). In addition, typical hematological parameters (white blood cell counts, red blood cell counts, hemoglobin levels, and hematocrit) and biochemical markers related to hepatic and renal functions were normalized to near-baseline levels following proKPV treatment (fig. S17). Daily proKPV administration for 7 days did not induce apparent systemic toxicity, as implicated by histological examination of hematoxylin and eosin (H&E)–stained sections of major organs and GI tissues (figs. S18 and S19). Consistent with these observations, proKPV demonstrated low cytotoxicity at concentrations ≤ 1000 μg/ml in RAW264.7 macrophages and Caco-2 epithelial cells after 24 hours of incubation (fig. S20).

In a separate study, we further compared in vivo efficacy of proKPV at 2.5 mg/kg and 5-aminosalicylic acid (5-ASA; a clinical first-line colitis drug) at 50 mg/kg in mice with DSS-induced colitis. The results demonstrated that proKPV exhibited superior therapeutic efficacy over 5-ASA, in terms of attenuating body weight loss, reducing disease activity scores, mitigating colon shortening, suppressing pro-inflammatory cytokine production, ameliorating oxidative stress, and restoring colon tissue integrity (fig. S21). Collectively, these results demonstrate that oral proKPV delivery achieves potent therapeutic outcomes in colitis mice, without detectable toxicity. It is worth noting that proKPV exhibited significantly potent efficacy compared to free KPV and 5-ASA at a nearly 20-fold high dose.

Mechanistically, oral therapy with proKPV significantly suppressed the expression of pro-inflammatory cytokines (TNF-α, IL-1β, and IL-6) and oxidative mediators [MPO, malondialdehyde (MDA), and ROS] in colonic tissues (Fig. 4, H to M). Consistently, immunofluorescence analysis revealed markedly elevated expression of Ly6G, CitH3, and NE in the model group, indicative of enhanced neutrophil infiltration and NET formation. proKPV treatment effectively reduced neutrophil accumulation and inhibited NETosis. Meanwhile, proKPV therapy promoted anti-inflammatory immune response, as evidenced by remarkably increased populations of M2 macrophages and regulatory T cells (T_reg_ cells). This shift was reflected in the notably up-regulated expression of CD206 (a M2 macrophage marker) and Forkhead box P3 (FOXP3, a T_reg_ cell marker) (Fig. 4N). These results demonstrate that proKPV therapy drives macrophage polarization from the pro-inflammatory M1 phenotype toward the anti-inflammatory M2 phenotype while enhancing T_reg_ cell differentiation. Increased M2 macrophages and T_reg_ cells in the colonic tissue are central to colitis repair by mediating synergistic anti-inflammatory and tissue repair effects. M2 macrophage–derived exosomes potentiate T_reg_ cell proliferation and IL-4 secretion through CCR8 axis activation, thereby suppressing pro-inflammatory cytokines (IL-1β, IL-6, and IL-17A) and promoting epithelial barrier restoration (*58*). In the lamina propria, CD206^+^ M2 macrophages maintain immune tolerance via IL-10 and transforming growth factor–β–mediated cross-talk with effector T_reg_ cells (*59*). This cellular interplay is further stabilized by the CCL1/CCR8 axis, which orchestrates T_reg_ migration and retention, whereas M2 macrophage–derived exosomes enhance T_reg_ suppressive capacity through Foxp3 and c-MAF up-regulation (*58*). These coordinated mechanisms collectively attenuate epithelial injury, stimulate goblet cell repopulation, and ultimately resolve mucosal inflammation and restore immune homeostasis. In all these aspects, free KPV administered at a dose equivalent to 10 mg/kg proKPV failed to elicit significant beneficial effects.

### Blood circulation kinetics, lung targeting, and therapeutic effects of orally delivered proKPV in ALI mice

To further validate the translational significance of our oral delivery platform, we extended therapeutic evaluations in a systemic disease model. Specifically, we assessed therapeutic efficacy of proKPV in mice with LPS-induced ALI, an inflammatory pulmonary disorder characterized by acute respiratory distress syndrome as its most severe clinical manifestation and remains a major contributor to morbidity and mortality among critically ill patients (*37*, *38*). Before therapeutic evaluations, we first investigated the blood circulation and pulmonary accumulation kinetics of proKPV NPs. Intravenous injection of Cy5-KPV in ALI mice resulted in a rapid elevation of plasma concentrations, which declined sharply to baseline levels within 4 hours (fig. S22). By contrast, plasma fluorescence signals in the oral Cy5-proKPV group increased gradually, peaking at 4 hours postadministration and remaining detectable up to 12 hours. Concomitantly, fluorescence signals in the oral Cy5-proKPV group progressively increased in the lungs of ALI mice (fig. S23). Notably, substantial fluorescence persisted in pulmonary tissues even at 12 hours following oral gavage. Conversely, Cy5-KPV signals were substantially attenuated by 6 hours after intravenous administration and became negligible by 12 hours. These findings demonstrate that oral administration of proKPV enables efficient intestinal barrier penetration, systemic absorption, and preferential targeting to inflamed lung tissues with prolonged retention.

**Fig. 5.**
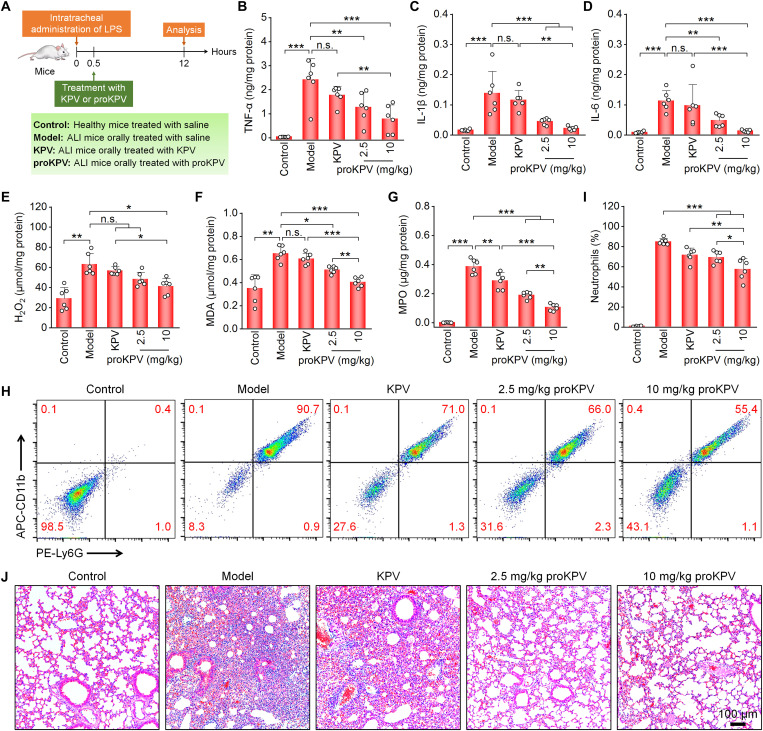
Therapeutic effects of orally delivered proKPV in ALI mice. (**A**) Schematic illustration of treatment regimens. (**B** to **G**) Levels of TNF-α (B), IL-1β (C), IL-6 (D), ROS (E), MDA (F), and MPO (G) in lung tissues isolated from healthy or diseased mice treated with different formulations. (**H** and **I**) Representative flow cytometric profiles and quantitative analysis of neutrophil counts in BALF of ALI mice. (**J**) H&E-stained lung tissue sections. Data in (B) to (G) and (I) are presented as means ± SD (*n* = 6 biological replicates). *P < 0.05; **P < 0.01; ***P < 0.001; n.s., not significant.

Subsequently, in vivo therapeutic effects of proKPV were evaluated. LPS-induced ALI mice were orally administered free KPV (at a dose equivalent to 10 mg/kg proKPV) or proKPV at 2.5 and 10 mg/kg (Fig. 5A). Treatment with proKPV, particularly at 10 mg/kg, significantly down-regulated levels of pro-inflammatory cytokines and oxidative mediators in lung tissues, including TNF-α, IL-1β, IL-6, H_2_O_2_, MDA, and MPO (Fig. 5, B to G). Consistent with the effective attenuation of pulmonary inflammatory and oxidative responses, oral administration of proKPV at both 2.5 and 10 mg/kg significantly reduced neutrophil counts in bronchoalveolar lavage fluid (BALF) (Fig. 5, H and I). Further histological evaluation of H&E-stained lung sections indicated that the ALI model group exhibited prominent inflammatory cell infiltration and alveolar hemorrhage, hallmark pathological features of ALI (Fig. 5J). Notably, these histological aberrations were markedly alleviated following proKPV treatment. By contrast, therapy with free KPV did not yield more potent therapeutic outcomes than oral proKPV at the tested two doses. Collectively, these findings confirm that oral delivery of proKPV can confer potent therapeutic effects in a mouse model of ALI, a representative systemic inflammatory disease.

### Engineering of amphiphilic peptide prodrug conjugates derived from Ac-QAW and IRW

**Fig. 6.**
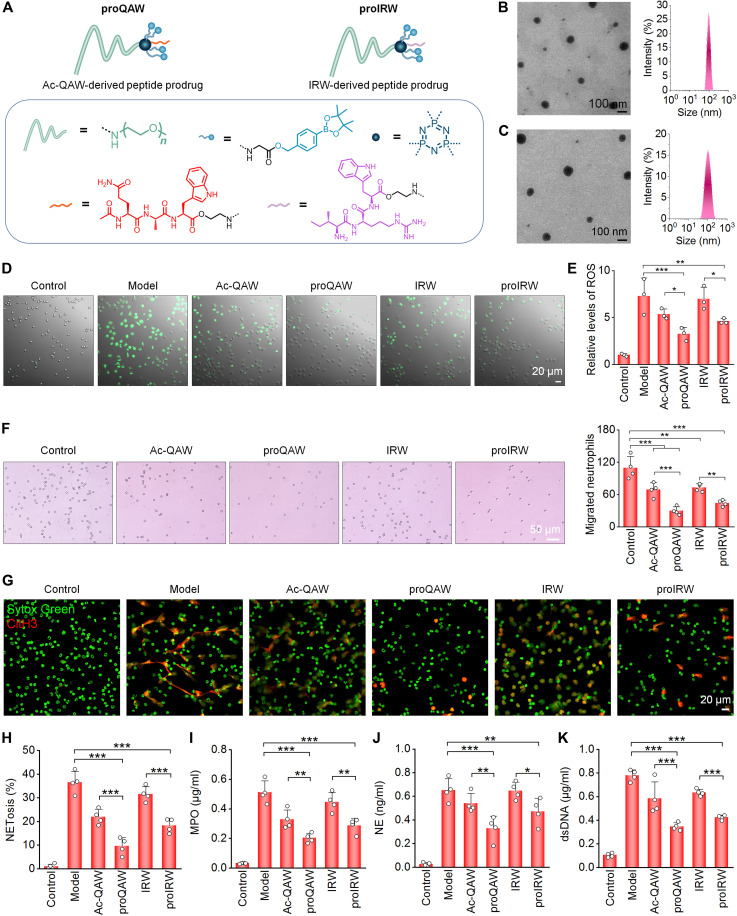
Synthesis, characterization, and in vitro biological activities of amphiphilic peptide conjugates derived from Ac-QAW or IRW. (**A**) Schematic illustration of chemical structures of peptide prodrug conjugates derived from Ac-QAW (proQAW) or IRW (proIRW). (**B** and **C**) TEM images (left) and size distribution profiles (right) of NPs assembled from proQAW (B) or proIRW (C). (**D** and **E**) Fluorescence microscopy images showing intracellular ROS generation (D) and flow cytometric quantification of relative ROS levels (E) in neutrophils following different treatments. (**F**) Optical microscopic images (left) and quantitative analysis (right) indicate migrated neutrophils after treatment with different formulations. (**G** and **H**) Representative confocal microscopy images (G) and quantitative analyses (H) of the NET formation after neutrophils were stimulated with PMA and treated with different formulations. (**I** to **K**) Levels of MPO (I), NE (J), and dsDNA (K) expressed by neutrophils after stimulation with PMA and treatment with different formulations. Data in (E), (F), and (H) to (K) are presented as means ± SD (*n* = 3 to 4 biological replicates). *P < 0.05; **P < 0.01; ***P < 0.001.

Building on the above promising results, we sought to evaluate the universality of our functional module prodrug approach for peptide delivery. To test the applicability, two anti-inflammatory peptides, i.e., Ac-QAW (Ac-Gln-Ala-Trp) and IRW (Ile-Arg-Trp), were used to synthesize the corresponding ROS-triggerable amphiphilic conjugates proQAW and proIRW, respectively (Fig. 6A and fig. S24). Ac-QAW, the N-terminal sequence of an anti-inflammatory protein annexin A1, inhibits the NF-κB (nuclear factor κB) signaling cascade to exert anti-inflammatory effects (*60*), whereas the egg-derived IRW peptide can attenuate TNF-α–induced inflammatory responses and oxidative stress (*61*). The chemical structures of proQAW and proIRW were confirmed by ^1^H NMR spectra (fig. S25). Both proQAW and proIRW can spontaneously self-assemble into micelle-like NPs upon dissolution in deionized water. DLS measurement revealed average diameters of 92.3 ± 3.3 nm for proQAW NPs and 83.7 ± 3.5 nm for proIRW NPs, with ζ-potential values of −0.07 and 0.6 mV, respectively (Fig. 6, B and C). Both NPs showed good stability in different solutions (fig. S26). The CMC was determined to be 43 and 107 μg/ml for proQAW and proIRW, respectively (fig. S27).

We next assessed biological activities of proQAW and proIRW in neutrophils. Confocal microscopy and flow cytometry analyses showed that pretreatment with proQAW and proIRW more effectively attenuated PMA-stimulated ROS generation in mouse peritoneal neutrophils compared to their free peptide counterparts (Fig. 6, D and E). Transwell migration assay further revealed that both peptide conjugates significantly inhibited neutrophil chemotaxis (Fig. 6F). Furthermore, we examined the impact of proQAW and proIRW treatment on the NET formation. Treatment of neutrophils with either proQAW, proIRW, or their corresponding free peptides (Ac-QAW and IRW) significantly suppressed PMA-induced NETosis, as evidenced by substantial reductions in both CitH3 expression and NETosis extent (Fig. 6, G and H). These findings were further corroborated by quantification of MPO, NE, and dsDNA release in PMA-activated neutrophils (Fig. 6, I to K). Notably, both conjugate prodrug formulations exhibited superior efficacy compared to free peptides, with proQAW showing particularly pronounced inhibitory effects.

### Therapeutic effects and safety profiles of proQAW and proIRW in colitis mice

**Fig. 7.**
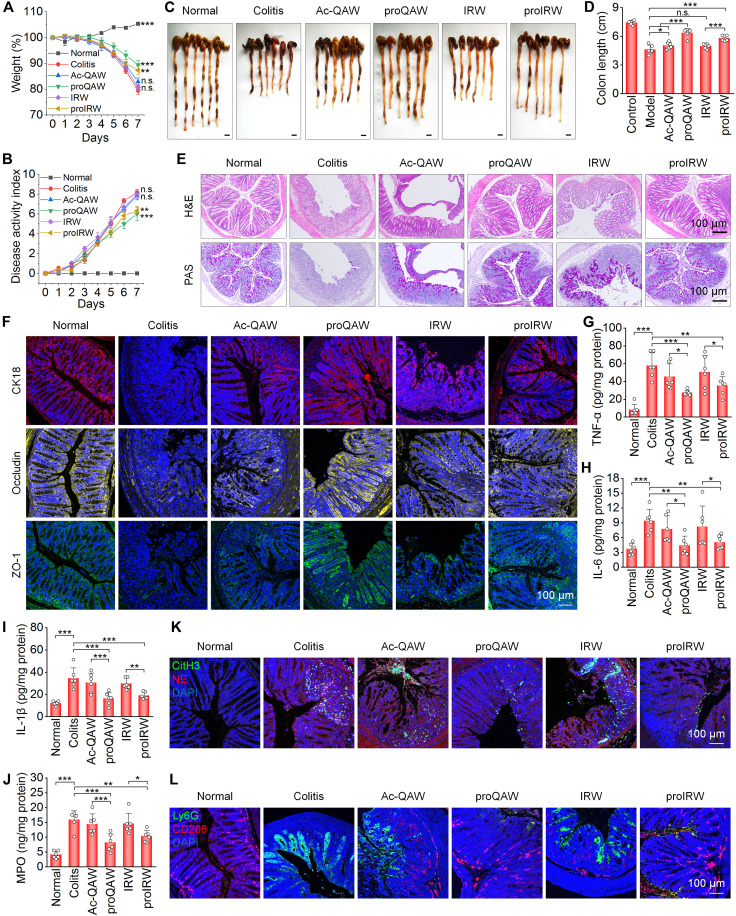
Therapeutic efficacy of proQAW and proIRW in mice with DSS-induced acute colitis. (**A**) Body weight changes of mice during 7-day treatment, which were normalized to the baseline at day 0. **P < 0.01 and ***P < 0.001; n.s., not significant versus the colitis group. (**B**) Changes in DAI values. **P < 0.01 and ***P < 0.001 versus the colitis group. (**C** and **D**) Digital photos (C) and quantified lengths (D) of colonic tissues isolated from mice after 7 days of treatment. (**E**) Histological sections of colonic tissues stained with H&E or PAS. (**F**) Immunofluorescence indicates expression patterns of CK18, occludin, and ZO-1 in colonic tissues. (**G** to **J**) Levels of TNF-α (G), IL-6 (H), IL-1β (I), and MPO (J) in colonic tissues isolated from healthy or diseased mice treated with different formulations. (**K** and **L**) Immunofluorescence analysis of NETs (K), Ly6G^+^ neutrophils, and CD206^+^ macrophages (L) in colonic tissues. Data in (A), (B), (D), and (G) to (J) are presented as means ± SD (*n* = 6 biological replicates). *P < 0.05; **P < 0.01; ***P < 0.001; n.s., not significant.

We then evaluated in vivo efficacy of proQAW and proIRW in mice with DSS-induced acute colitis (fig. S28). Oral administration of both prodrug conjugates ameliorated disease severity, as evidenced by attenuated weight loss, improved DAI, and preserved colon length compared to control groups (Fig. 7, A to D). Histopathological examination showed that prodrug-treated mice maintained intact colonic architecture with well-preserved crypts and goblet cells (Fig. 7E). PAS staining of colonic tissue sections confirmed abundant mucin production in these groups, whereas immunofluorescence analysis indicated high expression of epithelial barrier markers (CK18, occludin, and ZO-1), suggesting robust restoration of mucosal integrity (Fig. 7F). By contrast, treatment with free Ac-QAW or IRW showed minimal therapeutic effects. Furthermore, the prodrug conjugates effectively mitigated inflammatory and oxidative responses in colonic tissues, as indicated by significantly reduced levels of TNF-α, IL-6, IL-1β, MPO, H_2_O_2_, and MDA (Fig. 7, G to J, and fig. S29). Correspondingly, both proQAW and proIRW treatment markedly diminished neutrophil infiltration and NET formation, demonstrated by reduced expression of Ly6G, NE, and CitH3. Notably, the prodrugs promoted macrophage polarization toward the anti-inflammatory M2 phenotype, as indicated by elevated CD206 expression in colonic tissue sections (Fig. 7, K and L). Throughout all assessments, both peptide conjugates consistently outperformed their corresponding free peptide counterparts in therapeutic efficacy.

Moreover, preliminary safety evaluations revealed excellent biocompatibility profiles for both proQAW and proIRW. Following therapy with either proQAW or proIRW, no significant changes were found in the organ index of the heart, liver, spleen, lung, and kidney among different groups (fig. S30). Hematological analysis and serum biochemistry showed all parameters remained within normal physiological ranges (fig. S31). Histopathological examination of GI tissues from treated animals indicated intact mucosal architecture with no evidence of drug-induced lesions or inflammatory infiltrates (fig. S32), further confirming the safety of both peptide conjugates. Consistently, both proQAW and proIRW exhibited negligible cytotoxicity in RAW264.7 macrophages and Caco-2 epithelial cells at doses lower than 1000 μg/ml (fig. S33).

Together, these findings demonstrate that our developed peptide prodrug conjugates, i.e., proQAW and proIRW, exhibit potent therapeutic efficacy in treating acute colitis in mice, primarily by suppressing inflammatory cytokine production and oxidative stress, inhibiting neutrophil infiltration and NET formation, and facilitating macrophage polarization toward the anti-inflammatory M2 phenotype. Oral administration of these conjugates at therapeutic doses showed good safety profiles, with no observed systemic or GI toxicity. Although the free peptides (Ac-QAW and IRW) offered modest therapeutic effects, covalent conjugation into ROS-responsive self-immolative amphiphilic polymers notably enhanced their biological activity, mainly attributable to improved stability and targeted delivery to inflamed tissues.

## DISCUSSION

Anti-inflammatory peptides show great promise in managing various inflammation-associated disorders like IBD, acute lung injury, and sepsis (*39*, *62*, *63*), owing to their multifaceted biological properties, including anti-inflammatory and antioxidant capabilities, along with low toxicity. Oral administration represents the most convenient and suitable approach for treating localized inflammatory disorders in the GI tract (*64*–*66*). However, oral peptide bioavailability and bioactivity are typically limited by GI enzymatic degradation and poor penetration through biological barriers. Although enteric coatings effectively shield peptides from gastric degradation, they fail to prevent intestinal enzymatic hydrolysis. In addition, their pH-dependent release relies on variable physiological pH gradients along the GI tract, generally leading to erratic drug release profiles. Critically, such coatings cannot overcome the inherently poor epithelial permeability of peptides, frequently yielding low bioavailability. For currently used permeation enhancers (e.g., SNAC and sodium caprate), they exhibit narrow applicability and induce dose-limiting GI irritation in 10 to 15% of patients (*67*). Even formulations incorporating substantial permeation enhancers exhibit absolute oral bioavailability typically below 1%, with considerable interpatient variability (*19*, *68*). Additional limitations include uncertain safety profiles and narrow therapeutic indices (*69*, *70*). As for ingestible devices, their clinical translation remains constrained by complex fabrication protocols and cumbersome application procedures. Moreover, they have yet to demonstrate robust reliability or validated patient acceptance, particularly due to discomfort associated with the ingestion of macroscale objects. Systematic toxicological assessment following repeated dosing is imperative to address long-term safety concerns inherent to these delivery devices (*71*).

To overcome the limitations of oral peptide delivery, we developed a universal SIPPC strategy enabling targeted delivery of anti-inflammatory peptides to inflamed tissues. This involves the rational conjugation of anti-inflammatory peptides, a hydrophilic moiety PEG, and an inflammation-responsive hydrophobic module PBE onto a hydrolyzable cyclic phosphazene scaffold. The resulting amphiphilic peptide conjugates (derived from KPV, Ac-QAW, or IRW) can self-assemble into micelle-like NPs upon dissolving in aqueous solutions, incorporating peptide moieties into their cores. Following accumulation in inflamed tissues, these conjugates undergo complete hydrolysis into excretable and biocompatible hydrophilic compounds via a ROS-triggered self-immolative mechanism, thereby releasing peptides. The engineered peptide prodrug conjugates demonstrate resistance to hydrolysis in simulated gastric and intestinal fluids, preventing peptide degradation and premature release before reaching inflamed colonic tissues.

At the cellular level, anti-inflammatory peptide conjugates effectively suppressed macrophage and neutrophil migration and activation by mitigating inflammatory responses, inhibiting oxidative stress, and reducing NET formation. Following oral gavage, the peptide KPV-derived prodrug conjugate proKPV exhibited rapid and deep mucus penetration, accumulating notably in the injured colon of mice with DSS-induced acute colitis. It showed remarkable distribution in neutrophils and macrophages in colonic tissues. Consistently, proKPV achieved dose-dependent therapeutic effects in colitis mice, evidenced by significantly reduced weight loss and DAI, preserved colon length, and notably improved epithelial barrier function. Mechanistically, proKPV exerted its therapeutic efficacy by decreasing neutrophil recruitment, excessive activation, and NET formation, modulating macrophage polarization, and promoting T_reg_ cell differentiation in the colonic tissues.

In addition, using a mouse model of ALI, we further demonstrated that following oral gavage, proKPV effectively crossed the intestinal barrier, entered systemic circulation, and accumulated in inflamed lung tissues, thereby exerting potent dose-dependent anti-inflammatory effects. Moreover, the versatility of this SIPPC strategy was further validated with two additional anti-inflammatory peptides, i.e., Ac-QAW and IRW.

Compared with other peptide delivery formulations, our self-immolative prodrug conjugates own multiple advantages. First, this conjugation strategy enables high peptide loading capacity while facilitating straightforward formulation through direct dissolution in specific media, eliminating the need for complex formulation procedures. Second, covalent peptide-carrier conjugation prevents premature drug release and degradation during GI transit, ensuring conjugate integrity until reaching the target site. Furthermore, these conjugates exhibit both high therapeutic efficacy and safety profiles. Through site-specific hydrolysis in inflamed tissues, they generate water-soluble excretable metabolites while simultaneously releasing bioactive peptides, a dual mechanism that maximizes local therapeutic effects while minimizing systemic toxicity.

In summary, we established an inflammation-triggered self-immolative prodrug strategy for oral delivery of anti-inflammatory peptides. Conjugates rationally engineered via this principle not only enhance the physicochemical stability of peptides under harsh conditions but also enable site-specific accumulation in inflamed or injured tissues while achieving pathology-triggered smart peptide release. Besides validated oral delivery, these innovative peptide conjugates are suitable for injectable administration routes to treat diverse inflammatory disorders.

## MATERIALS AND METHODS

### Materials

HCCP, *N*-(*tert*-butoxycarbonyl)glycine (Boc-Gly), 4-(dimethylamino)pyridine (DMAP), 1,1′-carbonyldiimidazole (CDI), PBE, *N*,*N*′-dicyclohexylcarbodiimide (DCC), LPS from *Escherichia coli*, PMA, FITC-dextran (*M*_w_ = 4000 kDa), and 2′,7′-dichlorofluorescin diacetate (DCFH-DA) were supplied by Sigma-Aldrich (USA). KPV (Lys-Pro-Val), Boc-KPV, Cy5-KPV, Ac-QAW (Ac-Gln-Ala-Trp), IRW (Ile-Arg-Trp), and 9-fluorenyl methoxycarbonyl (Fmoc)–IRW were synthesized by ChinaPeptides Biological Technology Co. Ltd. (Shanghai, China). mPEG-Amine (PEG-NH_2_, *M*_w_ = 2000) was purchased from Laysan Bio Inc. (USA). Anhydrous dichloromethane (DCM), anhydrous tetrahydrofuran (THF), *N*,*N*′-diisopropylcarbodiimide (DIPC), triethylamine (TEA), and trifluoroacetic acid (TFA) were supplied by J&K Scientific Ltd. (Beijing, China). Cyanine5 NHS ester (Cy5-NHS) was supplied by Lumiprobe Co. Ltd. (USA). Dulbecco’s modified Eagle’s medium (DMEM), penicillin, streptomycin, and FBS were provided by Gibco (Waltham, USA). DSS (35 kDa) was purchased from MP Biomedicals (USA). 4′,6-Diamidino-2-phenylindole (DAPI), Amplex Red Hydrogen Peroxide/Peroxidase Assay Kit, SYTOX Green Nucleic Acid Stain, and Quant-iT PicoGreen dsDNA Reagent were obtained from Invitrogen (USA). Rabbit polyclonal anti-NE antibody and rabbit polyclonal anti-histone H3 (citrulline R2 + R8 + R17) antibody were obtained from Abcam (USA). All enzyme-linked immunosorbent assay (ELISA) kits were obtained from Boster Biological Technology Co. Ltd. (Wuhan, China). Oxidative stress–related kits and the BCA protein assay kit were obtained from Beyotime Biotechnology (Shanghai, China). The APC annexin V Apoptosis Detection Kit with propidium iodide (PI) was purchased from BioLegend (USA). All the other reagents were commercially available and used as received.

### Synthesis of Gly-PBE

Gly-PBE was synthesized as described in our previous study (*72*). Briefly, PBE (6.0 g), DMAP (0.32 g), and Boc-Gly (4.50 g) were dissolved in 200 ml of DCM. After incubation at 0°C for 1 hour, 5.3 g of DCC dissolved in DCM was added to the mixture, which was then warmed to room temperature. After stirring for 4 hours, the mixture was filtered and sequentially washed with 10% NH_4_Cl, 5% NaHCO_3_, and brine, followed by drying over Na_2_SO_4_. After drying under vacuum overnight, Boc-Gly–conjugated PBE (Boc-Gly-PBE) was obtained. Subsequently, 9.0 g of Boc-Gly-PBE was dissolved in a mixture of 40 ml of CF_3_COOH and CH_2_Cl_2_ at a volume ratio of 1:3 and stirred for 3 hours at room temperature before being poured into cold Et_2_O. The resulting precipitate was collected through filtration and dried overnight under vacuum. A white solid was yielded. ESI mass spectrometry was recorded on an ESI-Triple Quad mass spectrometer (Bruker). ^1^H NMR spectra were conducted via an Agilent DD2 spectrometer.

### Synthesis of PEG/PBE-conjugated HCCP

First, HCCP (710 mg) and TEA (616 μl) were dissolved in anhydrous DCM (10 ml), and then ethanolamine (EA) (123 mg) dissolved in 10 ml of anhydrous DCM was added dropwise under nitrogen protection. Afterward, the solution was stirred at −20°C for 3 hours. EA-conjugated HCCP (EA-HCCP) was obtained by purifying the mixture through chromatography on silica gel, resulting in a white solid. Subsequently, EA-HCCP (224 mg) and TEA (168 μl) were dissolved in 10 ml of anhydrous THF, into which mPEG-NH_2_ (1.0 g) was added dropwise at −20°C. After incubation at 0°C for 3 hours, a solution of Gly-PBE (1.05 g) and TEA (1 mL) in 10 ml of anhydrous THF was added dropwise to the reaction mixture and stirred at 75°C for another 48 hours under nitrogen protection. The mixture was then precipitated three times in cold ethyl ether. The isolated PEG/PBE-conjugated HCCP (PEP) was obtained after drying under vacuum overnight.

### Synthesis of proKPV

PEP (0.1 mmol), Boc-KPV (0.1 mmol), and DMAP (0.05 mmol) were dissolved in 5 ml of DCM. After 1 hour of reaction at 0°C, DIPC (0.1 mmol) was added and the mixture was stirred for another 24 hours at 25°C. Then, the mixture was poured into cold ethyl ether. The precipitate was collected by filtration, vacuum dried overnight, and subsequently subjected to Boc deprotection using TFA to obtain the final product proKPV. The obtained proKPV was validated by ^1^H NMR and FTIR spectroscopy (100S, PerkinElmer).

### Synthesis of proQAW and proIRW

PEP (0.1 mmol), Ac-QAW (0.1 mmol), and DMAP (0.05 mmol) were dissolved in 5 ml of DCM and stirred for 1 hour at 0°C. Then, DIPC (0.1 mmol) was added and the mixture was allowed to react for another 24 hours at 25°C. The reaction mixture was filtered and dialyzed against ethanol for 2 days. The obtained product was precipitated using cold ethyl ether, collected through filtration, and then vacuum dried overnight. For proIRW, Fmoc deprotection was conducted using 20% piperidine to obtain the final product.

### Synthesis of a fluorescent amphiphile of PCy5

PEP (0.1 mmol), Cy5-COOH (0.1 mmol), and DMAP (0.05 mmol) were dissolved in 5 ml of DCM and stirred for 1 hour at 0°C. Subsequently, DIPC (0.1 mmol) was added into the reaction mixture. After 24 hours, the mixture was filtered and dialyzed against ethanol for 2 days. The final product PCy5 was obtained by precipitation with cold ethyl ether, collected by filtration, and vacuum dried overnight.

### Measurement of CMC of different amphiphiles

The CMC of different conjugates in water was determined by the pyrene fluorescence method as described previously (*73*). Briefly, a series of amphiphile solutions with various concentrations was mixed with pyrene solution. After being heated at 50°C for 10 hours to equilibrate pyrene in micelles, the samples were cooled for 10 hours at room temperature and the final concentration of pyrene was 6.0 × 10^−7^ M. Excitation spectra were recorded on a fluorescence spectrophotometer (F7000, Hitachi High-Technologies Co., Tokyo, Japan) using an emission wavelength at 390 nm.

### Fabrication and characterization of peptide-derived NPs

proKPV NPs were prepared by directly dissolving proKPV in deionized water, followed by ultrasonication for 10 min. Subsequently, the resulting aqueous solution was statically equilibrated at room temperature for 30 min. Through similar procedures, proQAW NPs and proIRW NPs were fabricated. Measurements of particle size and ζ-potential were performed on a Malvern Zetasizer Nano ZS instrument at 25°C. TEM observation was conducted on a TECNAI-10 microscope (Philips, The Netherlands). To prepare the samples, the formvar-coated Cu grid was dipped into aqueous solutions of NPs for 5 min and stained with 1% phosphotungstic acid for 1 min.

### In vitro hydrolysis and release studies

In vitro hydrolysis of proKPV was performed at 37°C in aqueous solution with 10 mM hydrogen peroxide for 24 hours. The hydrolyzed products were collected by lyophilization and characterized by ^1^H NMR spectroscopy, ^31^P NMR spectroscopy, ESI mass spectrometry, and HPLC (LC-20A, Shimadzu).

For in vitro drug release tests, 1.0 ml of aqueous solution containing 10.0 mg of freshly fabricated proKPV was placed into dialysis tubing (MWCO: 8000 to 14000 Da), which was immersed in 40 ml of PBS (0.01 M, pH 7.4) with or without 1.0 mM hydrogen peroxide. At predetermined time intervals, 200 μl of release medium was withdrawn and replaced with an equal volume of fresh medium. The concentration of KPV in the release medium was quantified by HPLC. The detection wavelength was set at 220 nm, whereas the mobile phase comprised acetonitrile containing 0.1% TFA and aqueous solution containing 0.1% TFA (from 95 to 80% in 13 min), with a flow rate of 1.0 ml/min. Following similar procedures, drug release in media simulating the alimentary tract environment was also examined. Specifically, proKPV was first incubated in the SGF at pH 1.2 for 2 hours. Then, the dissolution medium was changed to PBS (0.01 M, pH 7.4) containing 1.0 mM hydrogen peroxide.

### Animals

Male C57BL/6J mice (20 to 24 g) and BALB/c mice (18 to 22 g) were supplied by the Animal Center of the Third Military Medical University. All animal experiments were performed in accordance with the Guide for the Care and Use of Laboratory Animals proposed by the National Institutes of Health. All procedures and protocols were approved by the Animal Ethics Committee at Third Military Medical University (no. AMUWEC20223362). Animals were housed in standard cages under suitable conditions, with ad libitum access to water and food. All mice were acclimatized for at least 7 days before further experiments.

### Cell culture

The RAW264.7 murine macrophage cell line was acquired from the Institute of Biochemistry and Cell Biology (Shanghai, China). To obtain neutrophils, male BALB/c mice were intraperitoneally injected with 1.0 ml of aqueous solution containing thioglycollate (3.0 wt %). After 4 hours, mice were euthanized and neutrophils were collected in peritoneal exudates by lavaging with sterile Hanks’ balanced salt solution (HBSS). Cells were cultured in DMEM containing 10% FBS with penicillin/streptomycin at 37°C in a humidified atmosphere containing 5% CO_2_.

### Stability of KPV and proKPV in physiological fluids

SGF [pH 1.2, containing pepsin (0.032 mg/ml)] and SIF [pH 7.4, containing trypsin (0.1 mg/ml)] were used for stability tests. Specifically, 100 μl of aqueous solution containing proKPV or free KPV (at 1 mg/ml KPV) was incubated at 37°C for 2 hours in 0.2 ml of SGF or SIF. Subsequently, the solution containing proKPV was filtered using ultrafiltration centrifuge tubes (3 kDa, Millipore) and 10 mM hydrogen peroxide was added to hydrolyze proKPV. The content of KPV was quantified by HPLC as aforementioned. In a separate study, 100 μl of aqueous solution containing proKPV was incubated at 37°C for 2 hours in 0.2 ml of hydrochloric acid at pH 2. The particle size and PDI were determined at defined time points. After 2 hours, the solution was lyophilized and analyzed by matrix-assisted laser desorption/ionization–time-of-flight (MALDI-TOF) mass spectrometry.

In another cohort study, 100 μl of aqueous solution containing proKPV or free KPV (at 20 μg/ml KPV) was incubated at 37°C for 2 hours in 0.2 ml of SGF or SIF. Then, the solutions containing proKPV were filtered using ultrafiltration centrifuge tubes, and the precipitate was collected and suspended in fresh medium. RAW264.7 macrophages were plated in 12-well plates at a density of 2 × 10^5^ cells per well and incubated overnight. After stimulation with LPS (100 ng/ml) for 6 hours, cells were cocultured with the solutions obtained above for an additional 4 hours. The supernatant was harvested, and the levels of TNF-α, IL-1β, and IL-6 were measured by the ELISA kit.

### In vitro cellular uptake

Neutrophils were cultured in glass-bottom cell culture dishes at a density of 5 × 10^5^ cells per well and incubated for 10 min. Subsequently, the culture medium was replaced with fresh medium containing Cy5-labeled proKPV NPs (Cy5-proKPV NPs; 50 μg/ml), which were prepared by mixing proKPV and PCy5 at a mass ratio of 10:1. After incubation for different time periods, nuclei were stained with DAPI and confocal laser scanning microscopy (CLSM) was used to capture fluorescence images. Similarly, dose-dependent internalization was analyzed after 1 hour of incubation.

Additional quantification of cellular uptake of proKPV NPs in neutrophils was performed via flow cytometry. Briefly, neutrophils were plated in 12-well plates at a density of 5 × 10^5^ cells per well. After 10 min, the culture medium was exchanged to 1 ml of fresh medium containing Cy5-proKPV NPs (50 μg/ml) and incubated for various durations. Then, the cells were collected and fluorescence intensities were measured by flow cytometry. Following similar procedures, dose-dependent uptake was examined after 1 hour of incubation.

### Studies of intracellular ROS generation in macrophages and neutrophils

To assess the inhibitory effect of proKPV, proQAW, or proIRW on intracellular ROS generation in PMA-activated macrophages, RAW264.7 cells were cultured in a 12-well plate (2 × 10^5^ cells per well) and incubated overnight. The cells were pretreated with different concentrations of NPs or free peptide for 2 hours at 37°C, and then the medium was replaced with PMA (200 ng/ml). After 1 hour of incubation, the cells were incubated with 10 μM DCFH-DA (a ROS-sensitive fluorescent probe) for 20 min. Subsequently, cells were harvested and fluorescence intensities were quantified by flow cytometry. For direct observation of ROS generation, RAW264.7 cells were cultured in confocal dishes at a density of 5 × 10^4^ cells per plate overnight. Following similar procedures as described above, fluorescence images were acquired by CLSM (Leica, Germany). Similar procedures were carried out to evaluate the inhibitory effect of NPs on intracellular ROS generation in PMA-stimulated peritoneal neutrophils.

### Apoptosis assay by flow cytometry

Apoptosis analysis was performed using the annexin V–APC and PI detection kit according to the manufacturer’s protocol. Specifically, RAW264.7 cells were seeded in a 12-well plate at a density of 2 × 10^5^ cells per well and incubated overnight. The medium was then replaced with fresh medium containing various formulations and incubated for 4 hours. Cells were treated with 400 μM H_2_O_2_ for another 6 hours. Afterward, cells were stained with the annexin V–APC conjugate and PI, and the fluorescence intensity was determined by flow cytometry. The staining patterns of annexin V^−^/PI^−^, annexin V^+^/PI^−^, and annexin V^+^/PI^+^ represent live cells, early apoptotic cells, and late apoptotic or necrotic cells, respectively.

### In vitro anti-inflammation tests

RAW264.7 macrophages were seeded in 12-well plates (2 × 10^5^ cells per well) and incubated overnight. After coculture with various NPs for 4 hours, cells were stimulated with LPS (100 ng/ml) for another 6 hours. The concentrations of TNF-α, IL-6, and IL-1β in the supernatant were determined by the ELISA kit.

### In vitro antimigration activity of various NPs in neutrophils

A transwell assay was conducted to examine antimigration activity of various NPs. In brief, isolated neutrophils (2 × 10^5^ cells) were incubated with various NPs or free peptides in the upper chamber of the transwell system. The lower chamber was filled with a FBS-free culture medium. Cells were allowed to migrate for 1 hour at 37°C, and then the cells in the lower chamber were observed and counted by optical microscopy.

### In vitro inhibition of the formation of NETs by NPs

To visualize the effects of NPs on the NET formation, neutrophils were seeded on sterilized glass coverslips in 12-well plates and preincubated with various NPs or free peptides for 1 hour. Subsequently, cells were stimulated with PMA for 4 hours, followed by washing three times with HBSS, and fixed with 4% paraformaldehyde for 1 hour. To observe citrullinated histones (CitH3), the fixed samples were permeabilized with Triton X-100 and blocked with bovine serum albumin. Then, the samples were incubated with a primary anti-CitH3 antibody (ab314916, Abcam, USA) and a secondary Cy3-conjugated rabbit-specific antibody (A0516, Beyotime, China), whereas dsDNA was stained with SYTOX Green dye.

In a separate study, neutrophils were seeded on sterilized glass coverslips in 12-well plates. Following similar procedures as described above, the levels of dsDNA or NETs in the medium were quantified by the Quant-iT PicoGreen dsDNA reagent, mouse elastase ELISA kit, or MPO ELISA kit according to the manufacturer’s protocols.

### Cytotoxicity of various peptide conjugates

To assess cytotoxicity of different NPs, RAW264.7 cells and Caco-2 cells were seeded in 96-well plates at a density of 1 × 10^4^ cells per well. Following 24 hours of incubation, the culture medium was replaced with 100 μl of fresh medium containing gradient concentrations of proKPV, proQAW, or proIRW, and the cells were further incubated for 24 hours. Cell viability was then quantified by the Cell Counting Kit-8 (CCK8) assay.

### Study on biodistribution of proKPV in mice with acute colitis by ex vivo imaging

Acute ulcerative colitis was induced in mice by drinking water containing 3% (w/v) DSS for 7 days (*50*). At day 8 after DSS treatment, mice received a single oral administration of Cy5-proKPV NPs (containing 3 μg of Cy5) or free Cy5-KPV, respectively. At defined time points, mice were euthanized. The colon segments, blood samples, and major organs (including the heart, liver, spleen, lung, and kidneys) were harvested. Ex vivo imaging was carried out with a living imaging system (In Vivo Imaging, Vilber, France). The fluorescence intensity was quantitatively analyzed using the Living Imaging software.

### Blood circulation kinetics and lung targeting of orally delivered proKPV in mice with ALI

ALI was induced in male BALB/c mice via intratracheal inoculation of 50 μl of PBS containing LPS (0.5 mg/ml) (*74*). Mice were then randomly assigned to two groups. After 0.5 hours post-LPS challenge, one group received oral gavage of Cy5-proKPV NPs (containing 6 μg of Cy5), whereas the other group received intravenous injection of Cy5-KPV. At predetermined time points, blood and lung samples were collected. Ex vivo imaging was performed using a living imaging system (Vilber, France), and fluorescence intensities were analyzed.

### Histological analysis of the cellular distribution of proKPV in the colon

The localization and cellular distribution of orally delivered NPs in the colon of colitis mice was also observed by CLSM to evaluate the selective accumulation of proKPV in the inflamed colon. Colitis mice were orally administered with Cy5-proKPV NPs or free Cy5-KPV as described above. After 6 hours, the colons were removed and embedded in Tissue-Tek O.C.T. Compound and sectioned at 7 μm thickness. The obtained cryosections were separately stained with anti–cytokeratin 18 (CK18) antibody (bsm-52058R, Bioss Biotechnology, China), anti-F4/80 antibody (30325, Cell Signaling Technology, USA), or anti-Ly6G antibody (31469, Cell Signaling Technology, USA) to further evaluate the tissue distribution of NPs. Then, the samples were incubated at 4°C overnight, and nuclei were stained with DAPI. Fluorescence images were captured after staining with Cy3-labeled secondary antibody (1:200; A0516, Beyotime, China).

### Multiple particle tracking analysis

Fresh mucus (200 μl) was obtained from the colon lumens of C57BL/6 mice and immediately incubated with PS NPs or Cy5-proKPV NPs (5 μl, 100 μg/ml) at 37°C for 30 min. Subsequently, the motion trajectories of PS NP or proKPV NPs in mucus were recorded by CLSM in the time series mode (frame rate: 37 fps, 5 min). R software and ImageJ were used to analyze motion characteristics of particles in mucus. The MSDs and *D*_eff_ values were calculated using the equations below

$$\text{MSD}={\left(x_{\left(t+\tau \right)}-x_{t}\right)}^{2}+{\left(y_{\left(t+\tau \right)}-y_{t}\right)}^{2}$$

$$D_{\text{eff}}=\text{MSD}/\left(\text{4τ}\right)$$

where τ represents the timescale, whereas *x* and *y* indicate the coordinates of the particles.

### Therapeutic effects of different peptide conjugates in mice with DSS-induced acute colitis

Acute ulcerative colitis in mice was induced by drinking water containing DSS as aforementioned. First, we examined therapeutic effects of proKPV after oral administration. In this case, colitis mice were randomly assigned to four groups (*n* = 6; each mouse was considered one independent biological replicate). Saline was orally administered in the model group, whereas free KPV at 1 mg/kg or different concentrations of proKPV (0.5 and 2.5 mg/kg) were orally administered daily during 7 days of DSS treatment. In addition, healthy mice treated with saline were used as the normal control. Mice were monitored daily, and changes in the body weight, visible stool consistency, and fecal bleeding were evaluated. DAI is defined as the summation of the fecal bleeding index (0 to 3), stool consistency index (0 to 3), and weight loss index (0 to 4) (*75*).

In an independent experiment, in vivo therapeutic efficacy of proKPV was compared with 5-ASA. Briefly, mice were randomly assigned to various groups. Healthy mice in the normal control group were not treated, whereas colitis mice were orally administered with saline, 5-ASA at 50 mg/kg, and proKPV at 2.5 mg/kg, respectively. Following the above mentioned procedures, therapeutic effects were assessed.

In another cohort study, animals were randomized to different groups. Healthy mice in the normal group were not treated, whereas colitis mice were orally treated with saline, Ac-QAW (0.25 mg/kg), proQAW (2.5 mg/kg), IRW (0.25 mg/kg), and proIRW (2.5 mg/kg), respectively. For all diseased mice, daily administration was performed during 7 days of DSS treatment. Then, similar evaluations were followed.

### Therapeutic evaluations in colitis mice after different treatments

After various treatments, mice were euthanized, and the whole colonic tissues (from the cecum to rectum) were separated. The colon length was measured. Subsequently, 1 cm of the distal colon was made into paraffin sections. The sections were stained with H&E or PAS. Also, colonic tissue cryosections were prepared and stained with anti-CK18 antibody (bsm-52058R, Bioss Biotechnology, China), anti-occludin antibody (27260-1-AP, Proteintech Biotechnology, China), anti–ZO-1 antibody (21773-1-AP, Proteintech Biotechnology, China), anti-Ly6G antibody (562737, BD Biosciences, USA), anti-CD206 antibody (ab64693, Abcam, USA), anti-CitH3 antibody (ab281584, Abcam, USA), anti-NE antibody (ab314916, Abcam, USA), or anti-FOXP3 antibody (ab215206, Abcam, USA). Subsequently, the slices were incubated with the secondary antibody, whereas nuclei were stained with DAPI. Then, cryosections were imaged by CLSM.

Moreover, the remaining colonic tissues were homogenized in cold PBS. After centrifugation, the levels of TNF-α, IL-1β, IFN-γ, MPO, and MDA in the supernatant were measured by ELISA, whereas the contents of H_2_O_2_ were measured by the Amplex Red Hydrogen Peroxide/Peroxidase Assay Kit. The concentration of soluble proteins was determined by the BCA protein assay.

In addition, major organs including the heart, liver, spleen, lungs, kidneys, stomach, and intestines were harvested, weighed, and processed into paraffin sections. After staining with H&E, histological sections were observed by optical microscopy. Blood samples were collected for quantification of biochemical markers relevant to liver/kidney functions and hematological parameters. In all these cases, analysis of endpoint readouts was carried out in a blinded manner. The investigators were blinded to group allocation during data analysis.

### Miniendoscopic imaging

An endoscopic video system for mice was used to directly visualize colonic mucosal damage. After various treatments, mice were anesthetized and the endoscopic procedure was viewed and digitally recorded on a triple chip camera (Karl Storz, Germany).

### Epithelial permeability assay

After 7 days of various treatments for colitis mice, each mouse received FITC-dextran by oral gavage at 0.5 mg/g. Blood samples were collected and centrifuged after 4 hours. The supernatant was diluted with PBS (1:3, v/v), and the FITC fluorescence was measured (excitation at 485 nm and emission at 535 nm).

### Therapeutic effects of orally delivered proKPV in mice with ALI

ALI was induced in mice as described. Mice were randomized into four groups (*n* = 6 per group; each as an independent biological replicate). At 0.5 hours post-LPS stimulation, the model group received oral saline, and other groups were treated by oral gavage with proKPV (2.5 or 10 mg/kg) or free KPV (equivalent to 10 mg/kg proKPV). Healthy mice in a normal control group received saline. At 11 hours posttreatment, mice were euthanized. BALF was collected, and neutrophil counts were analyzed by flow cytometry. In a parallel experiment, lung tissues were paraffin embedded, sectioned, stained with H&E, and observed microscopically. The remaining lung tissues were homogenized. Levels of TNF-α, IL-1β, IL-6, H_2_O_2_, MDA, and MPO were measured using the aforementioned methods.

### Statistical analysis

Data are expressed as means ± SD. For experiments involving multiple groups, statistical analysis was conducted using a one-way analysis of variance (ANOVA) test, whereas an unpaired *t* test was used for data with two groups. A value of *P* < 0.05 was considered statistically significant. No data were excluded from the analyses in this study.
