# Supplementary material for: Inflammation-triggered self-immolative conjugates enable oral peptide delivery by overcoming gastrointestinal barriers
Source: Sci Adv. 2026 Jan 14;12(3):eaea2989. doi: 10.1126/sciadv.aea2989 (PMC12802832; doi:10.1126/sciadv.aea2989)
Supplement: Supplementary file 1 — Figs. S1 to S33 [file sciadv.aea2989_sm.pdf]

Science Advances
AAAS

Supplementary Materials for

Inflammation-triggered self-immolative conjugates enable oral peptide delivery by overcoming gastrointestinal barriers

Juan Cheng *et al.*

Corresponding author: Chenwen Li, lichenwen@tmmu.edu.cn; Sheng Chen, chenshengerk@163.com; Jianxiang Zhang, jxzhang@tmmu.edu.cn, jxzhang1980@gmail.com

Sci. Adv. **12**, eaea2989 (2026)
DOI: 10.1126/sciadv.aea2989

This PDF file includes:

Figs. S1 to S33

Supplementary Figures

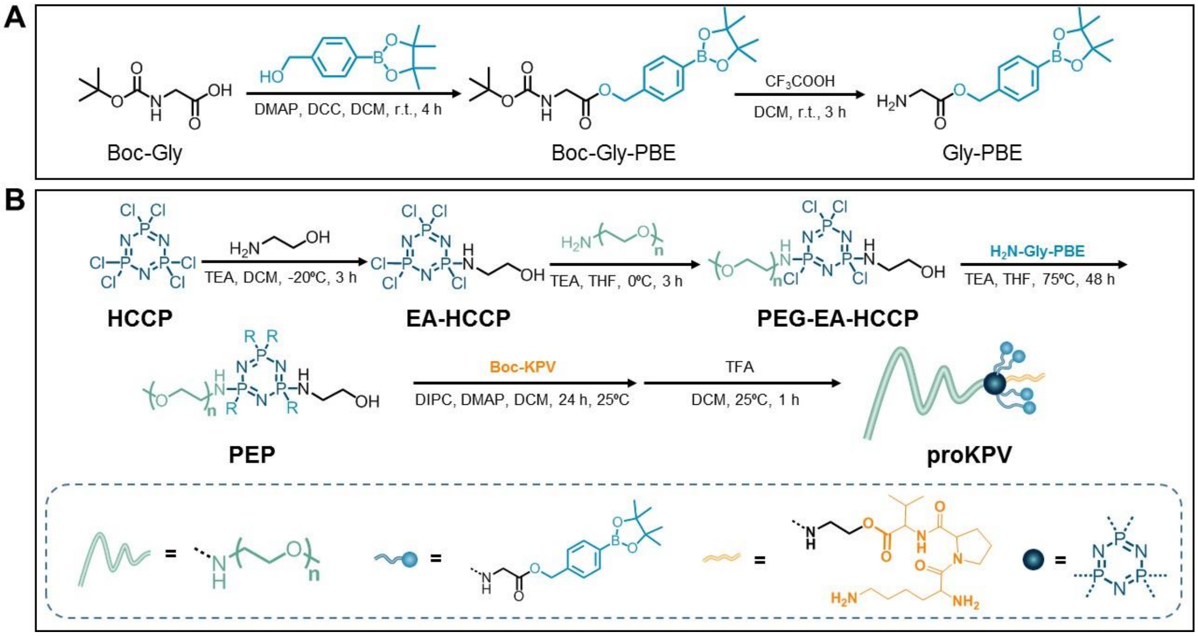

Fig. S1. The synthetic route of proKPV. (A) Synthesis of Gly-PBE by the esterification reaction and N-Boc deprotection. (B) Synthesis of proKPV. The synthesis proceeds through a sequential conjugation process: HCCP is initially conjugated with ethanolamine (EA), followed by PEG-NH_2 conjugation through subsequent nucleophilic substitution reactions. Gly-PBE is then coupled to the EA/PEG-functionalized HCCP intermediate. The final step involves KPV conjugation through esterification between Boc-protected KPV and the EA moiety on EA/PEG-HCCP. Boc-Gly, N-(tert-Butoxycarbonyl) glycine; DMAP, 4-dimethylaminopyridine; DCC, N, N′-dicyclohexylcarbodiimide; DCM, anhydrous dichloromethane; DIPC, N,N'-diisopropylcarbodiimide; PBE, 4-(hydroxymethyl) phenylboronic acid pinacol ester; HCCP, hexachlorocyclotriphosphazene; TEA, Triethylamine; THF, anhydrous tetrahydrofuran; TFA, trifluoroacetic acid; r.t., room temperature.

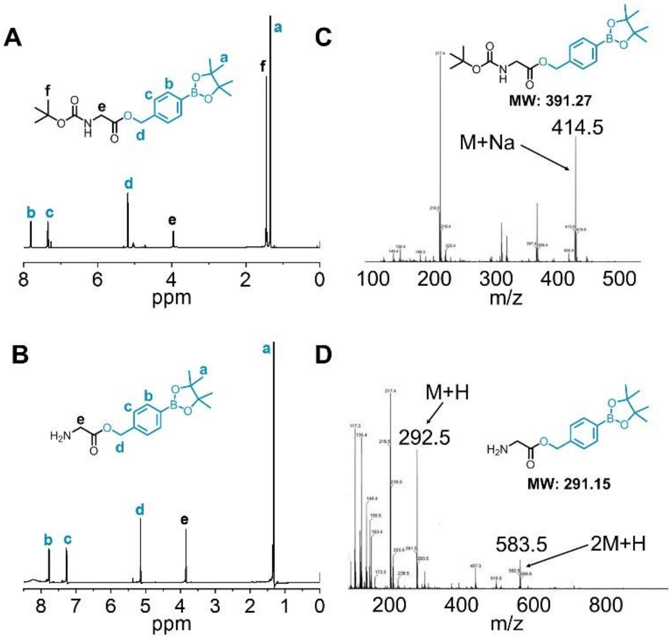

Fig. S2. Characterization of Boc-Gly-PBE and Gly-PBE. (A-D) ^{1}H NMR spectra (A-B) and ESI mass spectra (C-D) of Boc-Gly-PBE (A, C) and Gly-PBE (B, D).

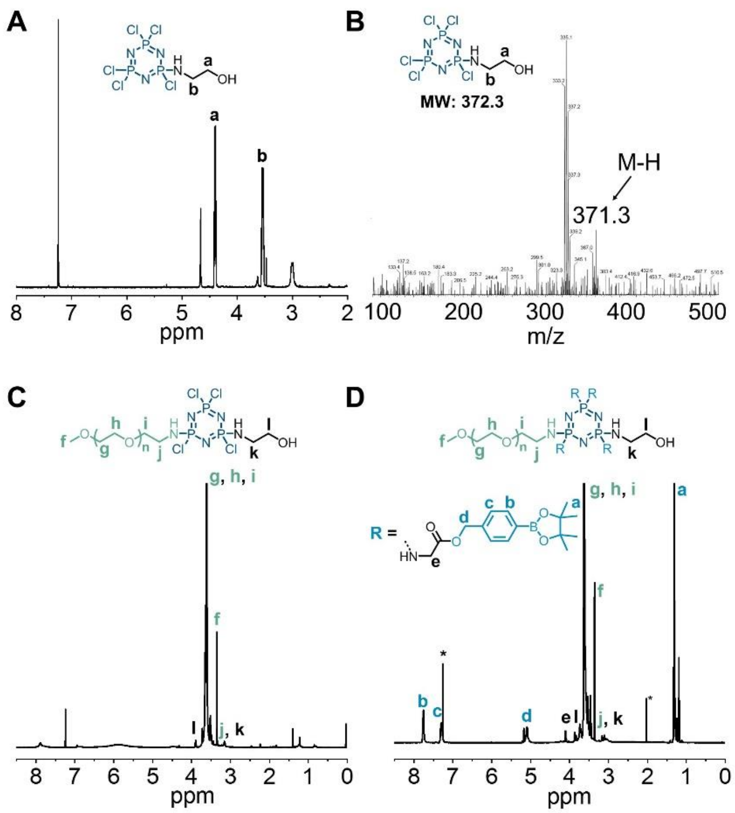

Fig. S3. Characterization of different intermediates. (A-B) ^{1}H NMR spectrum (A) and ESI mass spectrum (B) of EA-HCCP. (C-D) ^{1}H NMR spectra of PEG-EA-HCCP (C) and PEP (D).

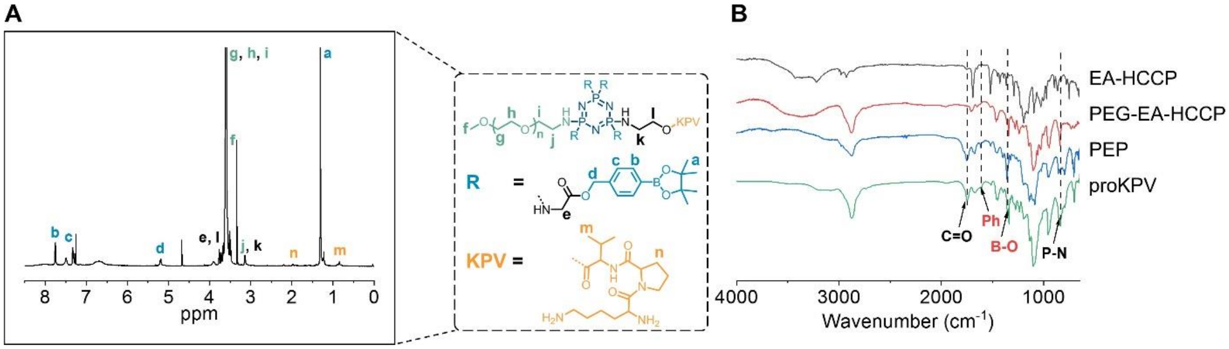

Fig. S4. Characterization of proKPV. (A-B) ^{1}H NMR (A) and FTIR (B) spectra of proKPV.

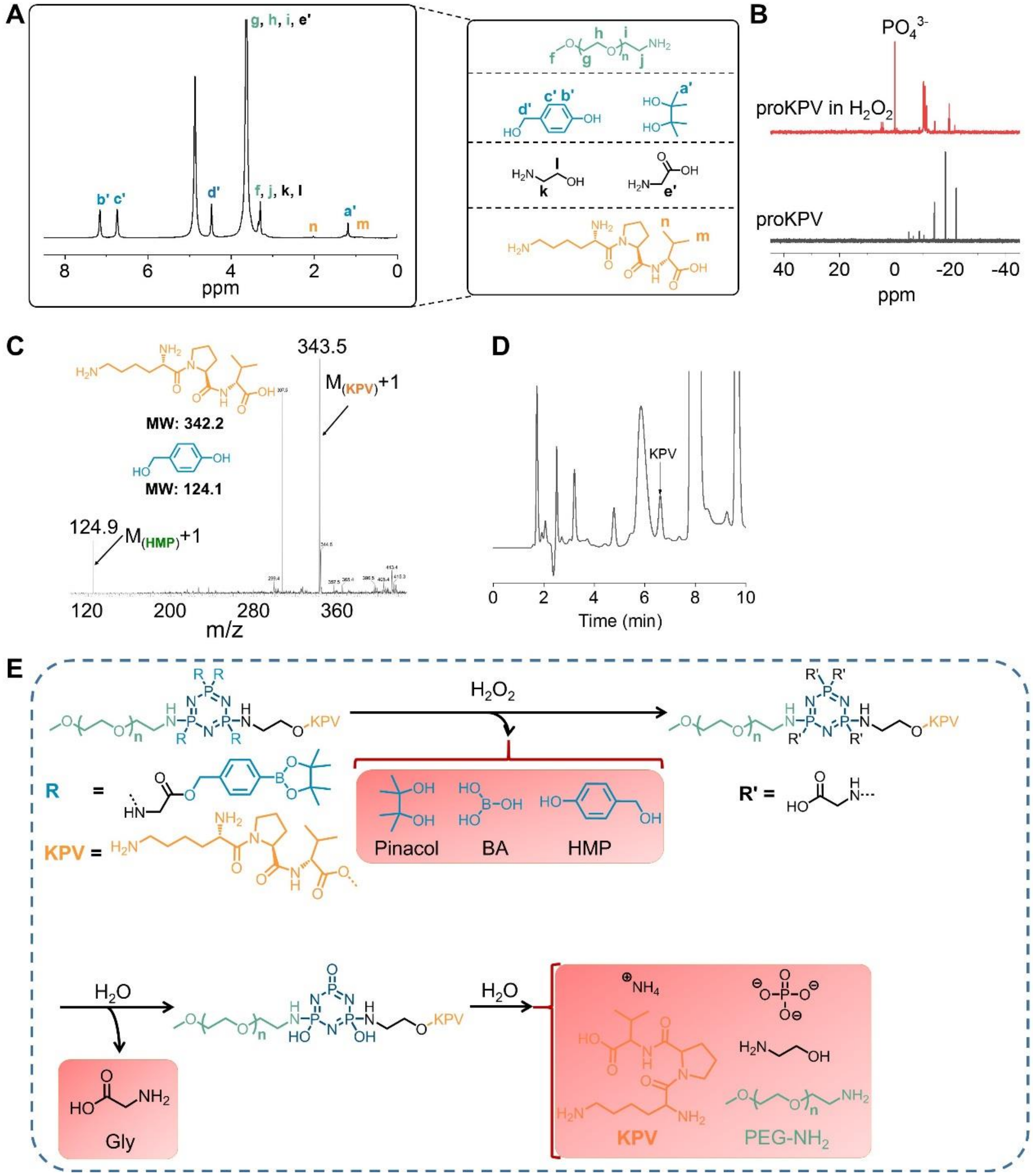

Fig. S5. ROS-triggered hydrolysis of proKPV. (A) ^{1}H NMR spectrum of proKPV in CD_3OD after incubation with H_2O_2. (B) ^{31}P NMR spectra of proKPV before and after H_2O_2-mediated hydrolysis. (C) ESI mass spectrum of the hydrolyzed products of proKPV. (D) HPLC detection of KPV after proKPV was incubated in 1.0 mM H_2O_2. (E) Hydrolysis mechanisms of proKPV in the presence of H_2O_2. BA, boric acid; Gly, glycine; HMP, p-(hydroxymethyl)phenol.

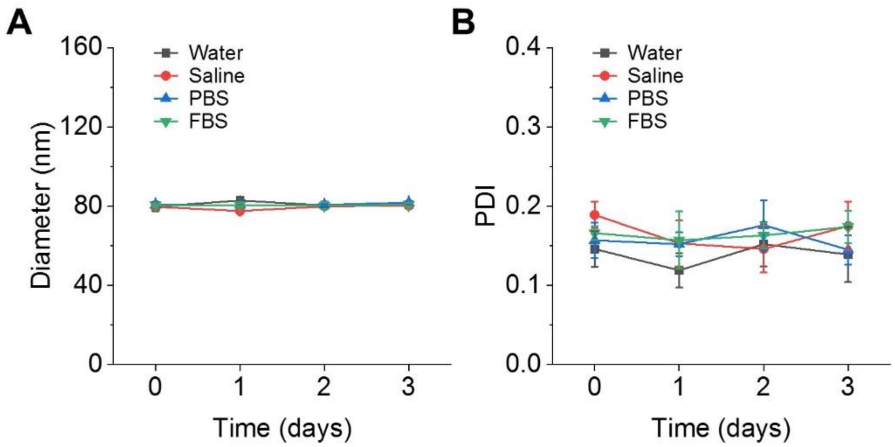

Fig. S6. Stability of proKPV in different solutions. (A-B) Time-dependent changes in the mean diameter (A) and polydispersity index (PDI) (B) of proKPV NPs after incubation in different solutions. Data are presented as mean ± SD (n = 3).

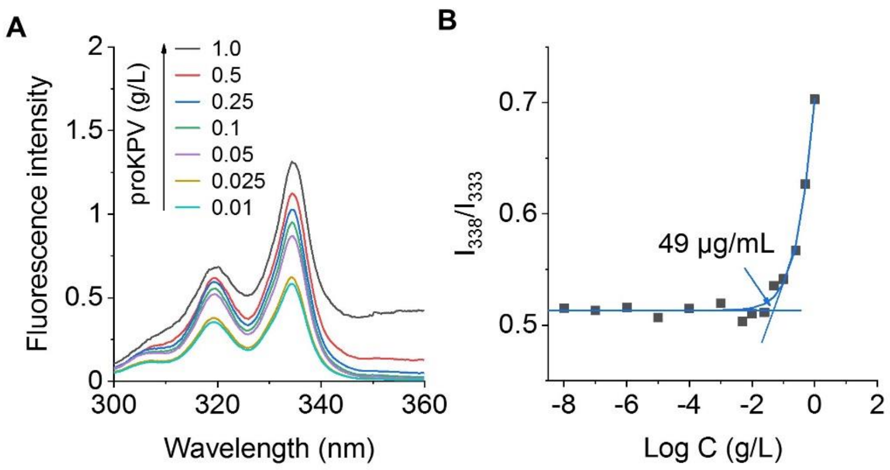

Fig. S7. Characterization of micellization behaviors of proKPV by a pyrene-based fluorescence probe method. (A-B) Fluorescence excitation spectra of pyrene in aqueous solutions containing different concentrations of proKPV (A) and the corresponding plot of the I_{338}/I_{333} band intensity ratio as a function of logarithmic concentration (B).

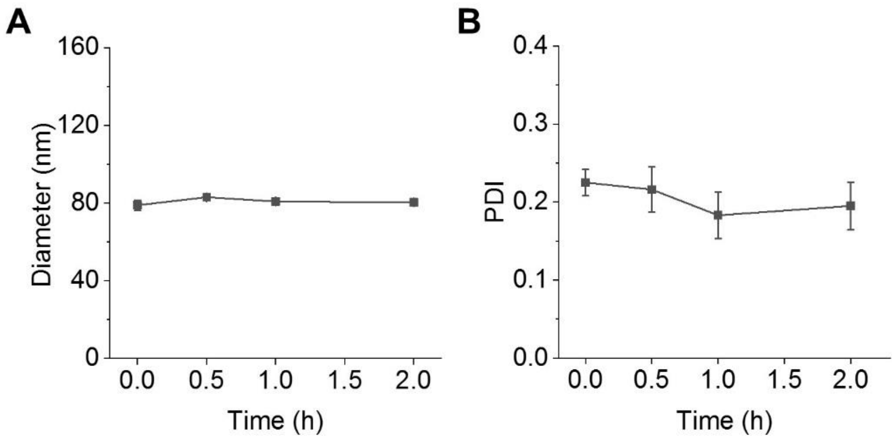

Fig. S8. Stability of proKPV NPs in hydrochloric acid at pH 2. (A-B) Time-dependent changes in the mean diameter (A) and PDI (B) of proKPV NPs after incubation in hydrochloric acid at pH 2 for 2 h. Data are presented as mean ± SD (*n* = 3).

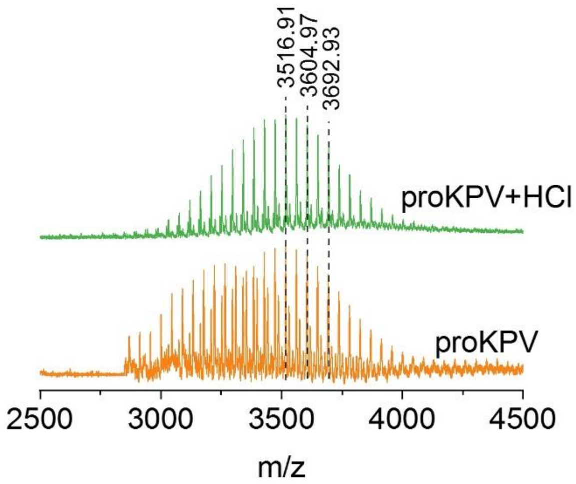

Fig. S9. MALDI-TOF mass spectra of untreated proKPV and proKPV treated in hydrochloric acid at pH 2.

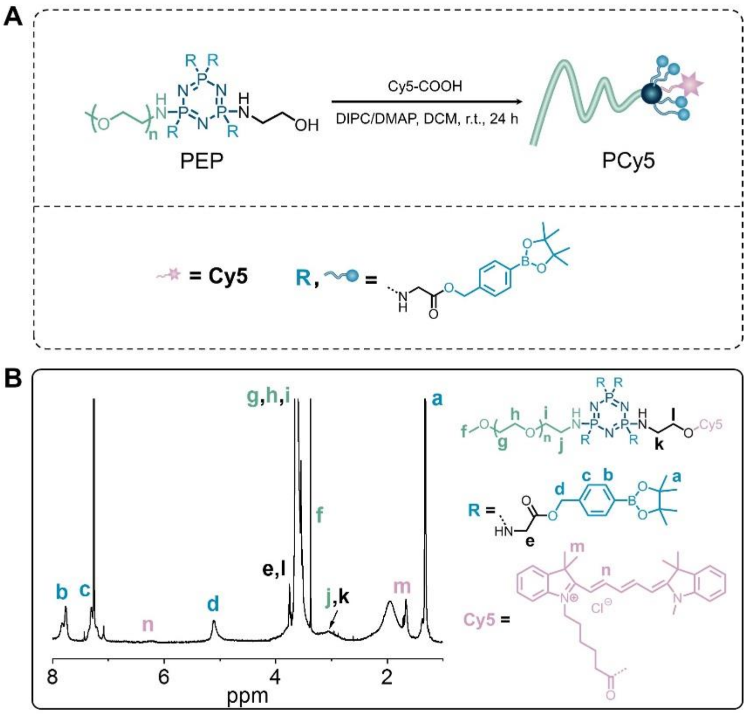

Fig. S10. Synthesis and characterization of a Cy5-labeled conjugate (PCy5). (A-B) The synthetic route (A) and ^{1}H NMR spectrum of PCy5 (B).

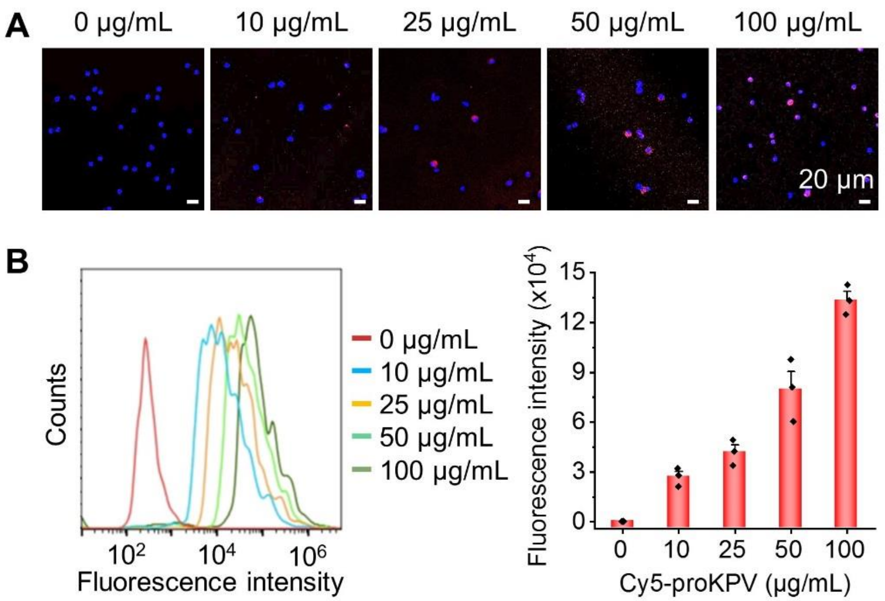

Fig. S11. Dose-dependent cellular uptake of Cy5-labeled proKPV NPs in neutrophils. (A) Fluorescence images showing dose-dependent internalization of Cy5-proKPV NPs at various doses after 1 h of incubation in mouse neutrophils. Nuclei were stained with DAPI. (B) Typical flow cytometric profiles (left) and quantification results (right) indicating cellular uptake of Cy5-proKPV NPs at various doses after 1 h of incubation in neutrophils. Data are mean ± SD (n = 3 biological replicates).

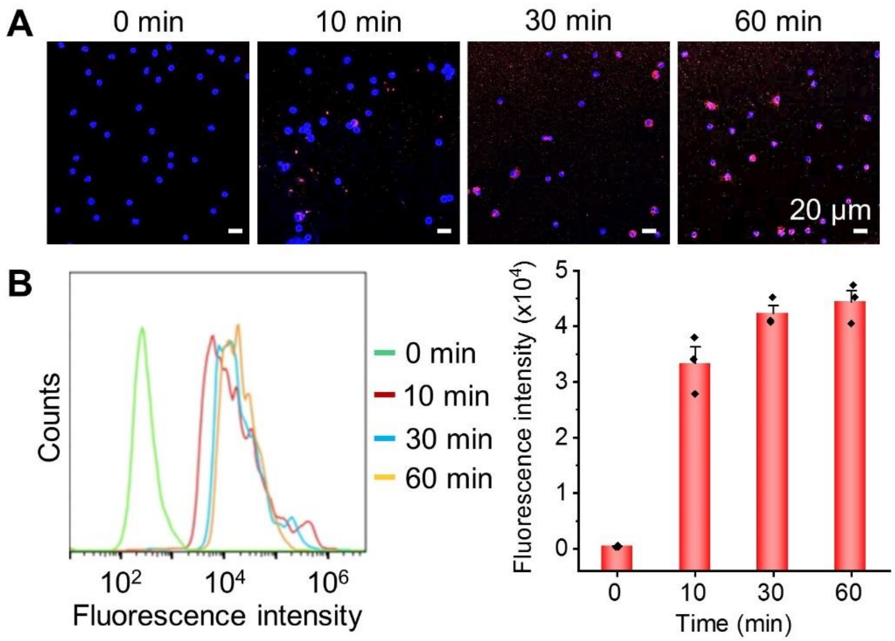

Fig. S12. Time-dependent cellular uptake of Cy5-proKPV NPs in neutrophils. (A) Fluorescence images showing time-dependent internalization of Cy5-proKPV NPs at 50 μg/mL in mouse neutrophils. Nuclei were labeled with DAPI. (B) Typical flow cytometric curves (left) and quantitative analysis (right) of time-dependent cellular uptake of 50 μg/mL Cy5-proKPV NPs in neutrophils. Data are mean ± SD (*n* = 3 biological replicates).

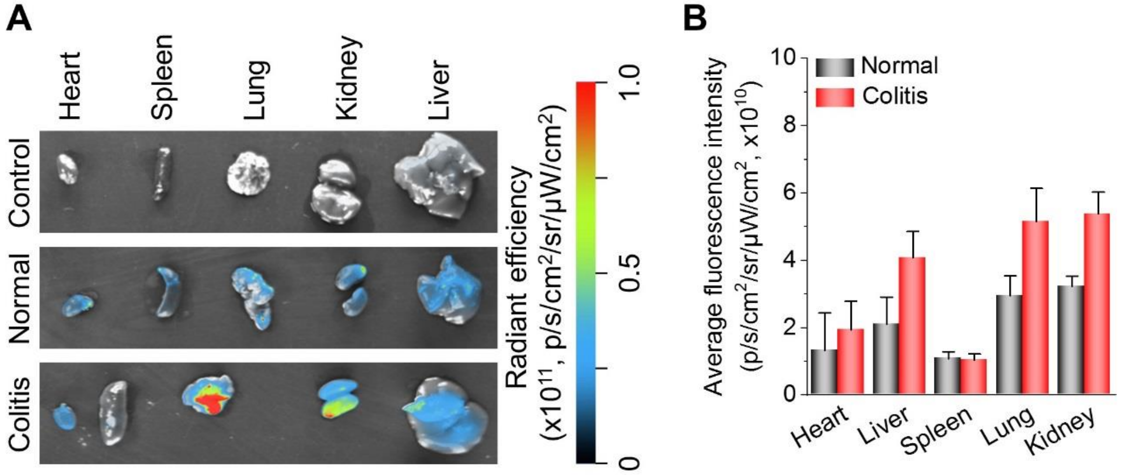

Fig. S13. Biodistribution of Cy5-proKPV NPs in major organs following oral gavage in mice. (A-B) Ex vivo fluorescence images (A) and quantified fluorescence intensities (B) illustrate the distribution of Cy5-proKPV NPs in major organs including the heart, liver, spleen, lung, and kidneys. Healthy mice (the normal control) and DSS-induced colitis mice were orally administrated with Cy5-proKPV NPs (containing 3 μg Cy5). At 6 h post-administration, major organs were isolated for ex vivo imaging. In the control group, mice were orally administrated with saline. Data are presented as mean ± SD (n = 3 biological replicates).

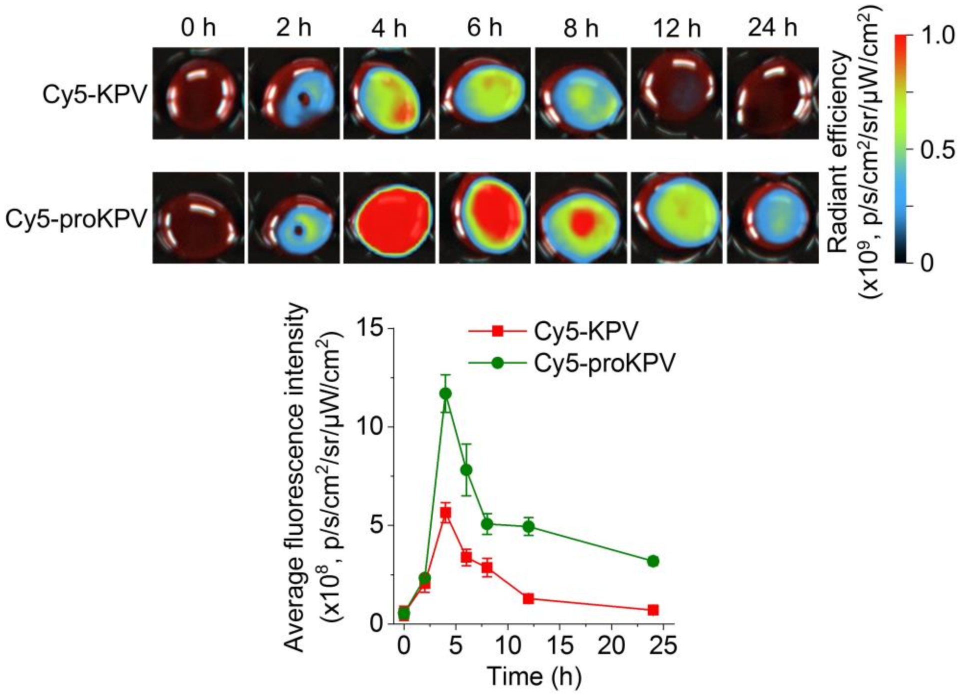

Fig. S14. Representative fluorescence images (upper) and quantitative analysis (lower) of blood samples from colitis mice treated with free Cy5-KPV or Cy5-proKPV by oral gavage. Data are presented as mean ± SD (*n* = 3 biological replicates).

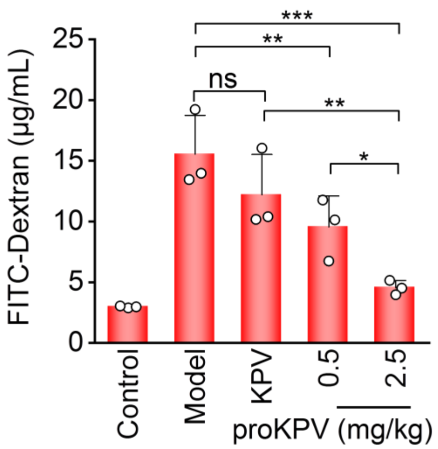

Fig. S15. Evaluation of intestinal barrier integrity of colitis mice following different treatments. Mice with DSS-induced acute colitis were treated with different formulations for 7 days. The barrier function was assessed at day 7. Each mouse was orally treated with FITC-Dextran at 0.5 mg/g. Blood samples were collected at 4 h after administration. The permeability of intestinal epithelium was evaluated by measuring the fluorescence intensities. Data are expressed as mean ± SD (*n* = 3 biological replicates). $^{*}P < 0.05$, $^{**}P < 0.01$, $^{***}P < 0.001$; ns, no significance.

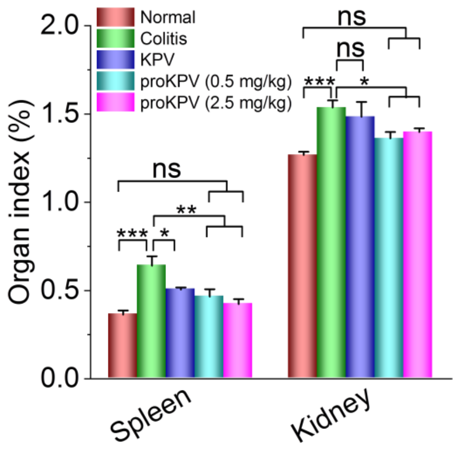

Fig. S16. The organ index values of the spleen and kidney isolated from mice of different groups. Data are expressed as mean ± SD (n = 6 biological replicates). $^{*}P < 0.05$, $^{**}P < 0.01$, $^{***}P < 0.001$; ns, no significance.

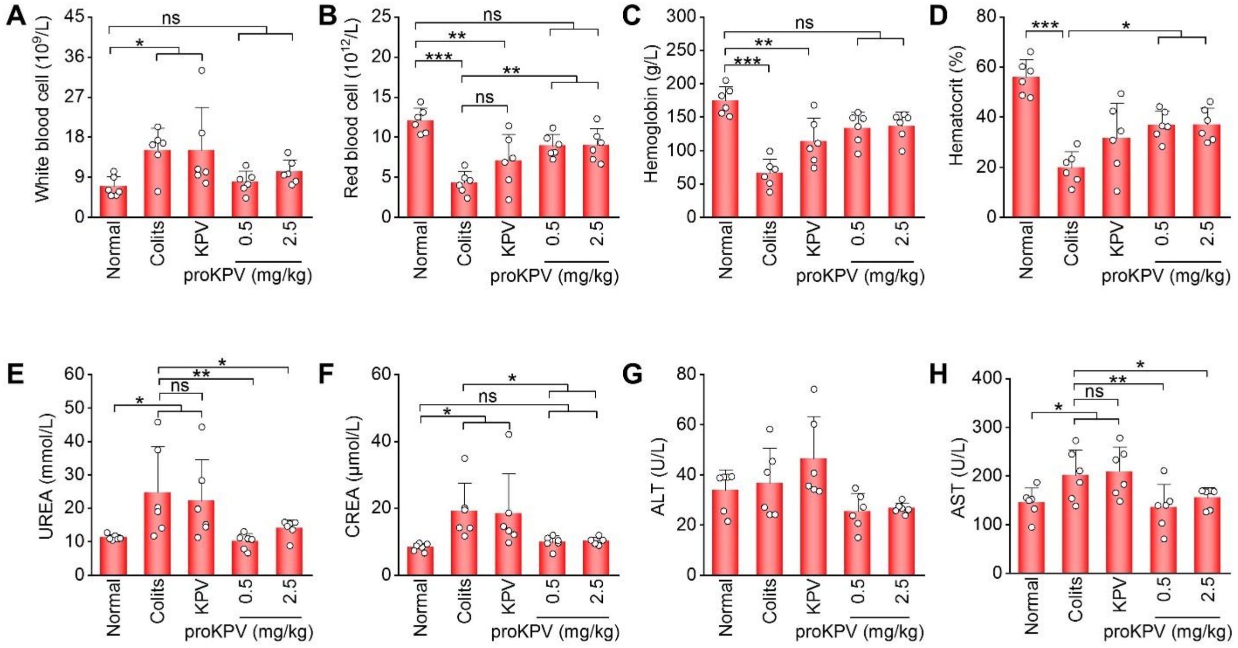

Fig. S17. Quantitative data of typical hematological parameters and biochemical markers relevant to hepatic and kidney functions in mice after different treatments. (A-D) Levels of white blood cells (A), red blood cells (B), hemoglobin (C), and hematocrit (D). (E-H) The levels of UREA (E), CREA (F), ALT (G), and AST (H). UREA, blood urea; CREA, creatinine; ALT, alanine transaminase; AST, aspartate transaminase. Data are expressed as mean ± SD (n = 6 biological replicates). $^{*}P < 0.05$, $^{**}P < 0.01$, $^{***}P < 0.001$; ns, no significance.

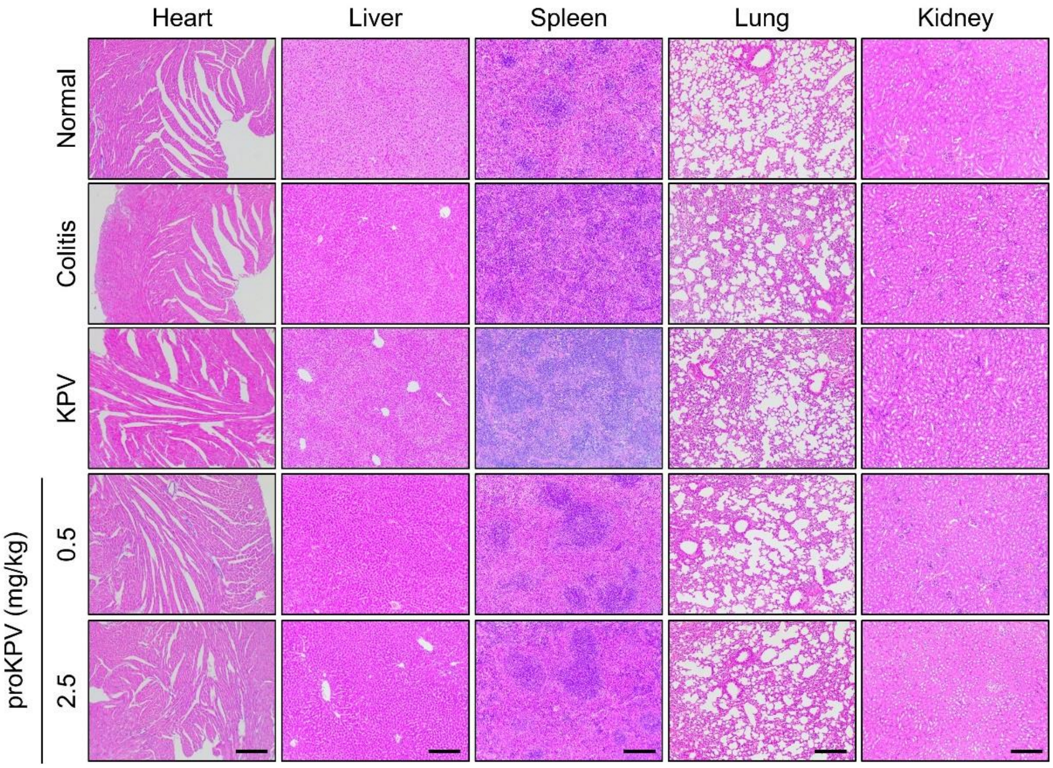

Fig. S18. Histological evaluations of major organs from mice after different treatments. Scale bars, 100 μm.

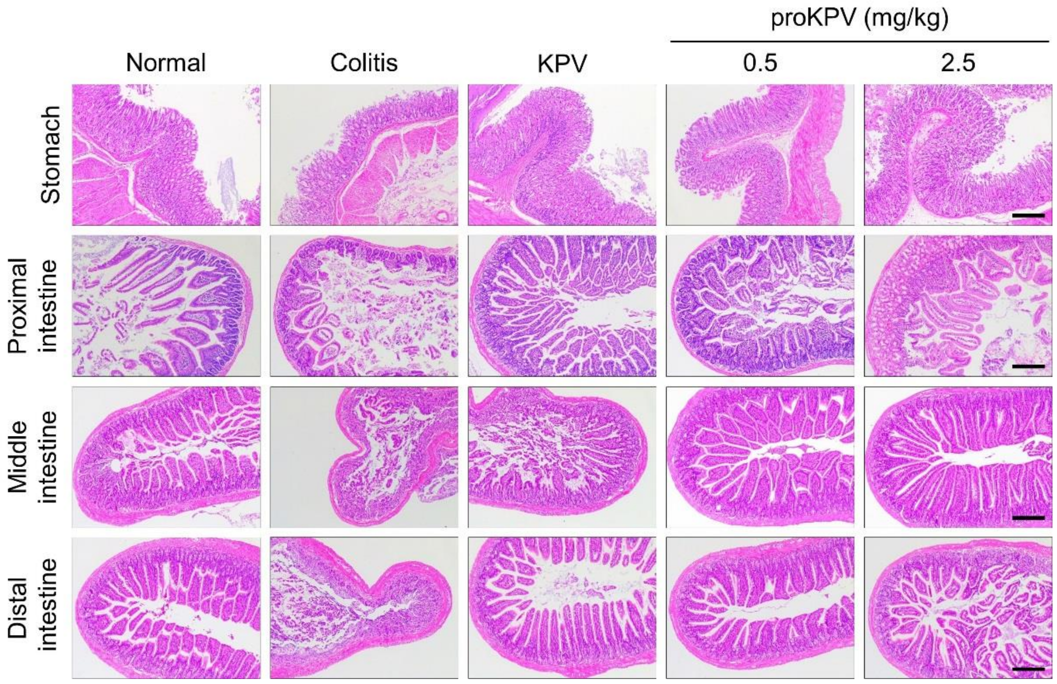

Fig. S19. H&E-stained histological sections of gastrointestinal tissues from mice with DSS-induced acute colitis after treatment with different formulations. Scale bars, 100 μm.

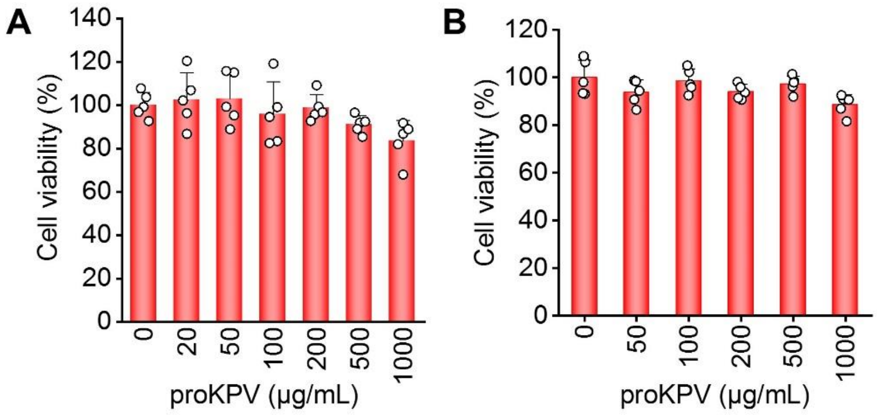

Fig. S20. Cytotoxicity evaluation of proKPV in different cell lines. (A-B) Cell viability of RAW264.7 macrophages (A) and Caco-2 cells (B). After 24 h of incubation with various doses of proKPV, cell viability was quantified by CCK8. Data are presented as mean ± SD (*n* = 5).

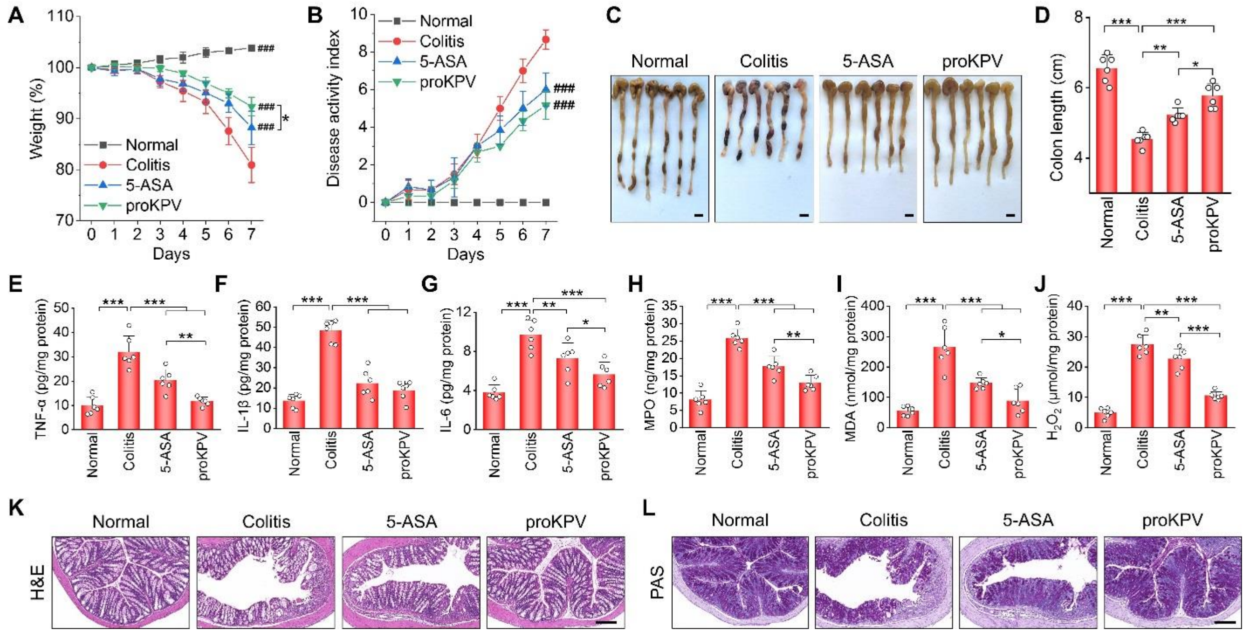

Fig. S21. Comparison of therapeutic effects of orally delivered proKPV and 5-ASA in mice with DSS-induced acute colitis. (A) The body weight of mice during a 7-day treatment period. Data were expressed as percentages relative to the body weight recorded on day 0. $^{###}P < 0.001$ versus the colitis group. (B) Changes in DAI. $^{###}P < 0.001$ versus the colitis group. (C-D) Digital photos (C) and quantified lengths (D) of colons obtained from mice on day 7 after receiving different treatments. Scale bars, 5 mm. (E-J) The levels of TNF-α (E), IL-1β (F), IL-6 (G), MPO (H), MDA (I), and ROS (J) in colonic tissues isolated from healthy or diseased mice treated with different formulations. Following 7 days of treatment, colonic tissue homogenates were prepared, and mediator concentrations were determined and normalized to the total protein content. (K-L) Histopathological evaluation of colon sections stained with H&E (K) or PAS (L). Data in (A-B, D, E-J) are presented as mean ± SD (n = 6 biological replicates). $^{*}P < 0.05$, $^{**}P < 0.01$, $^{***}P < 0.001$; ns, no significance. Scale bars, 100 μm.

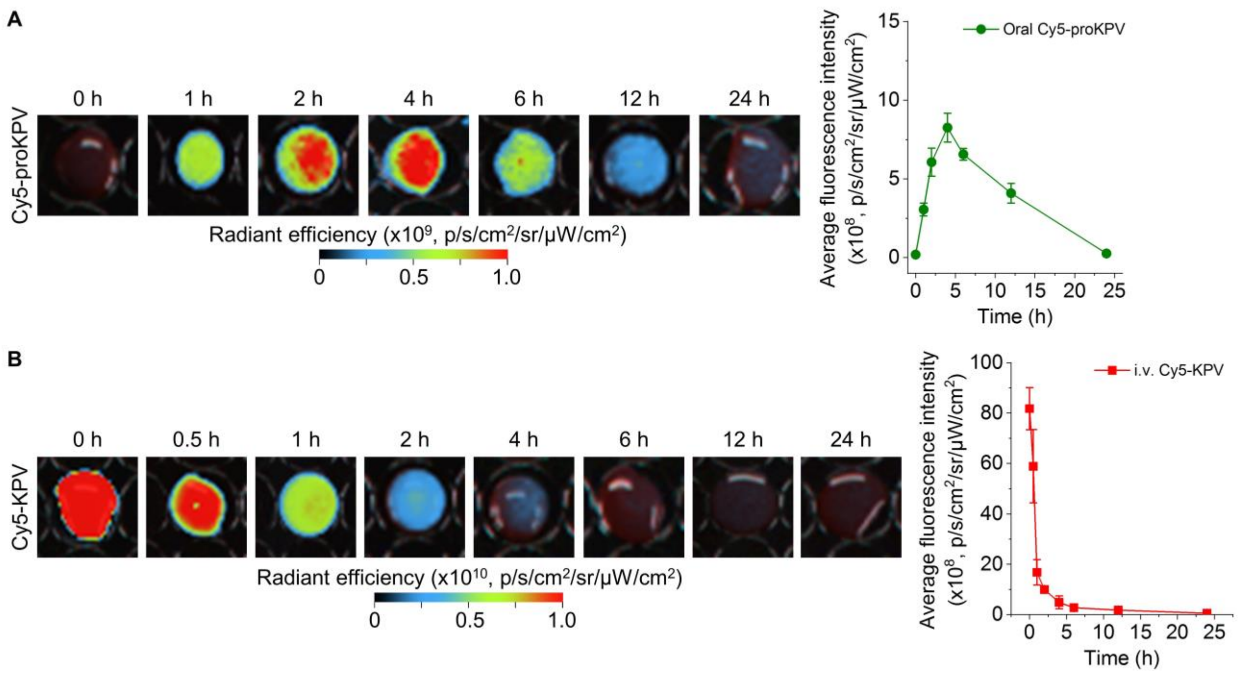

Fig. S22. Blood circulation kinetics of Cy5-proKPV or free Cy5-KPV in mice with acute lung injury (ALI). (A-B) Fluorescence images (left) and quantitative analysis (right) of blood samples from ALI mice following oral administration of Cy5-proKPV (A) or i.v. injection of Cy5-KPV (B). Data are presented as mean ± SD (*n* = 3 biological replicates).

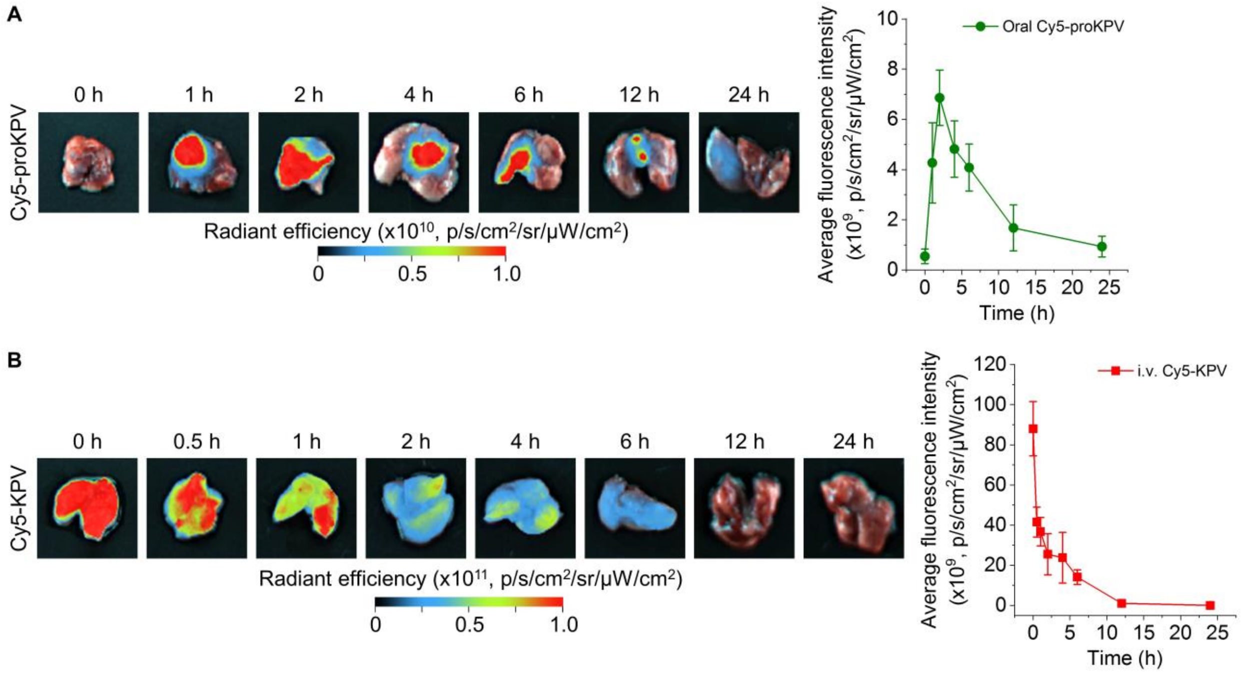

Fig. S23. Inflamed lung accumulation of Cy5-proKPV or free Cy5-KPV in ALI mice. (A-B) Fluorescence images (left) and quantitative analysis (right) of lung tissues from ALI mice following oral administration of Cy5-proKPV (A) or i.v. injection of Cy5-KPV (B). Data are presented as mean ± SD (n = 3 biological replicates).

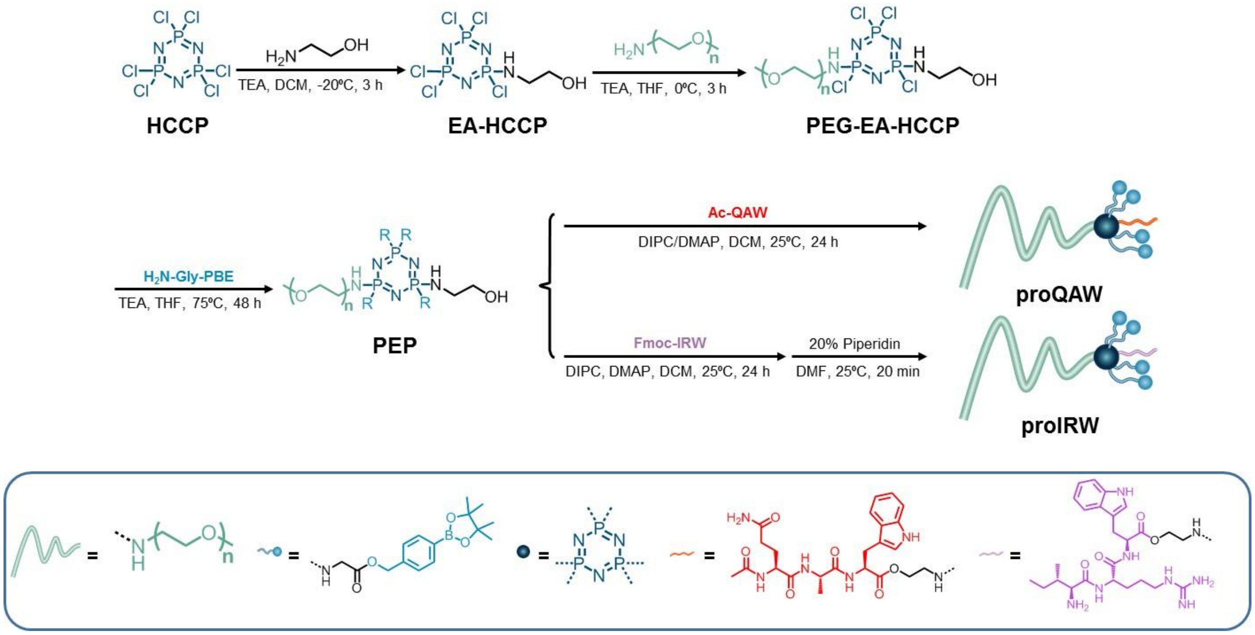

Fig. S24. Synthetic routes for proQAW and proIRW.

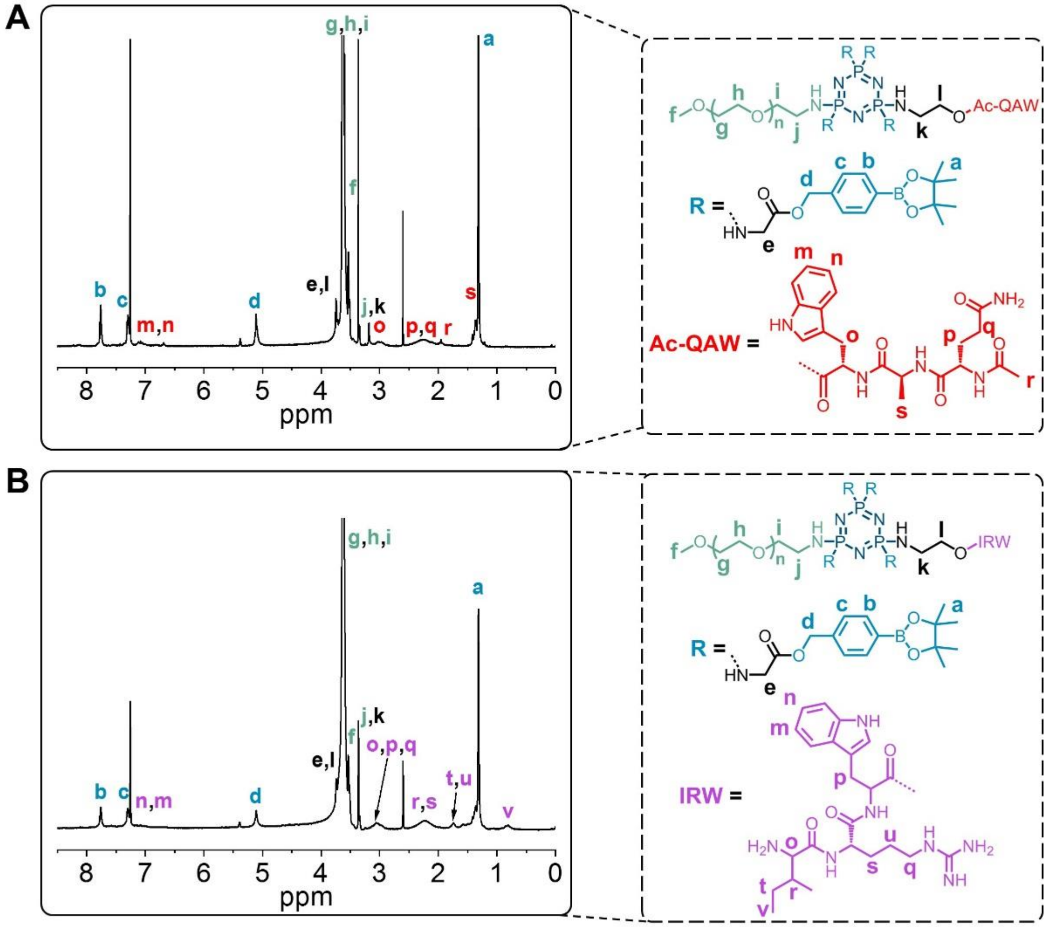

Fig. S25. Spectroscopy characterization of proQAW and proIRW. (A-B) ^{1}H NMR spectra of proQAW (A) and proIRW (B).

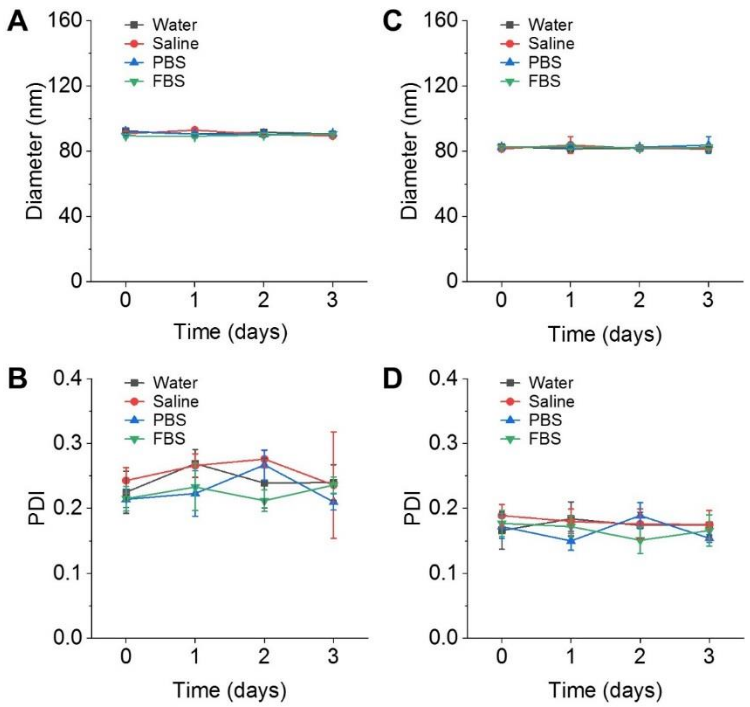

Fig. S26. Stability of proQAW NPs and proIRW NPs in different solutions. (A-D) Time-dependent changes in the mean diameter and PDI of proQAW NPs (A-B) and proIRW NPs (C-D) following incubation in various solutions. Data are presented as mean ± SD (n = 3).

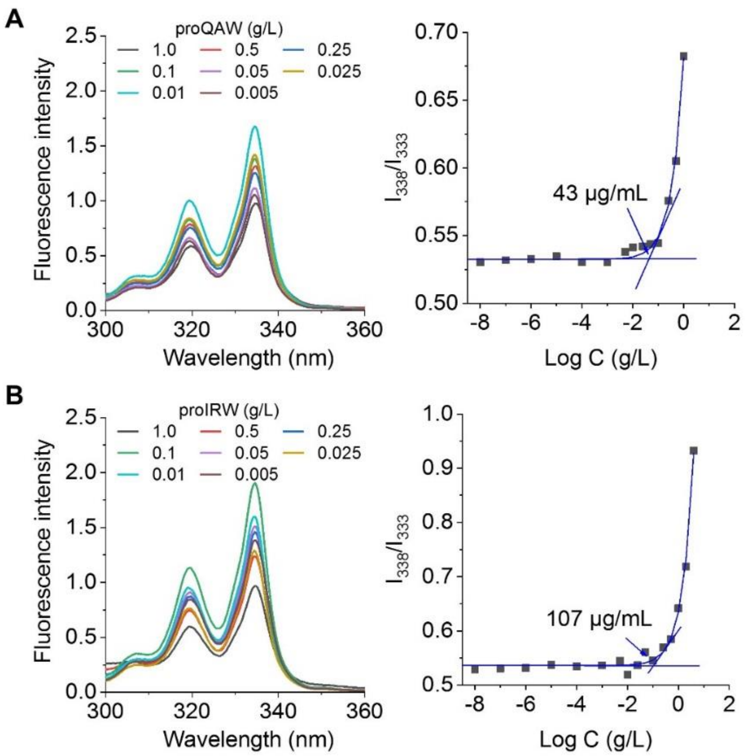

Fig. S27. Characterization of micellization behaviors of proQAW and proIRW by a pyrene-based fluorescence probe method. (A-B) Fluorescence excitation spectra (left) of pyrene in aqueous solutions containing different concentrations of proQAW (A) or proIRW (B) and the corresponding plots (right) of the I_{338}/I_{333} band intensity ratio as a function of logarithmic concentration.

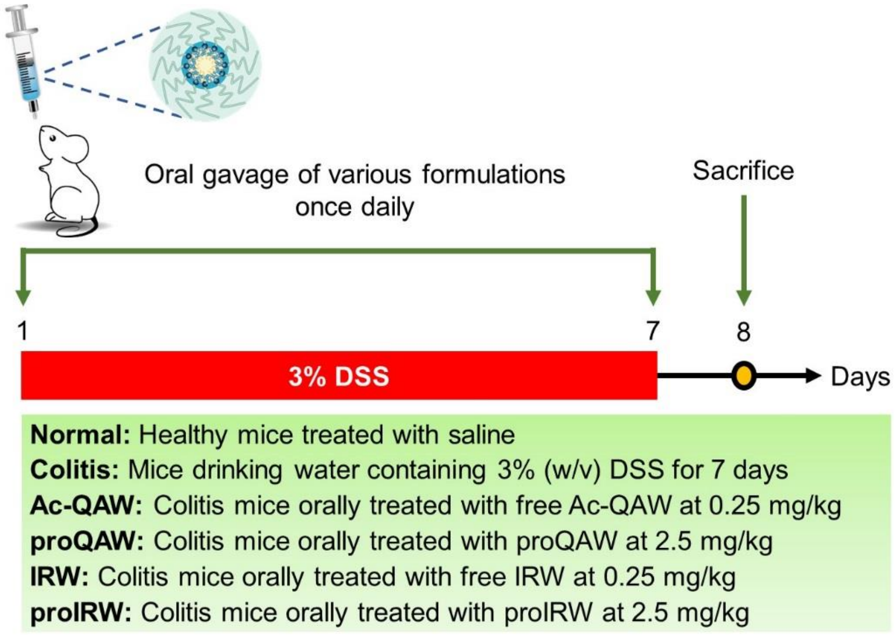

Fig. S28. Schematic diagram showing treatment regimens for therapeutic evaluations of proQAW and proIRW in mice with DSS-induced colitis.

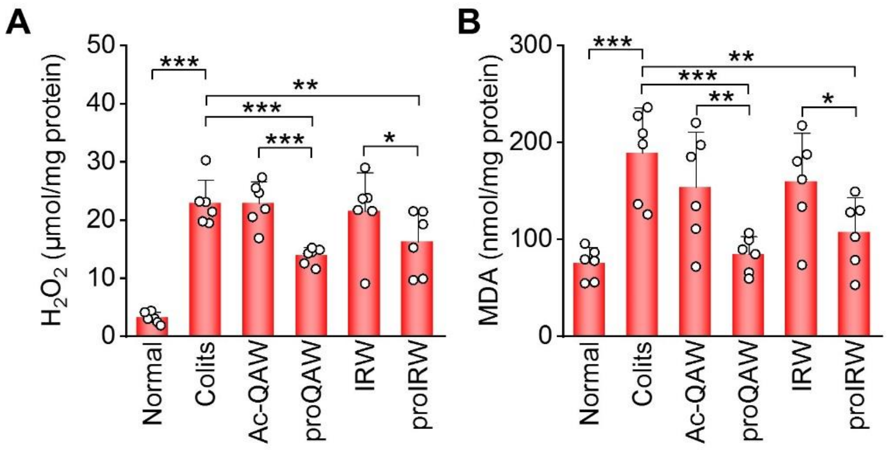

Fig. S29. Antioxidant effects of proQAW and proIRW in mice with DSS-induced acute colitis. (A-B) The levels of H_2O_2 (A) and MDA (B) in colonic tissues isolated from healthy or diseased mice treated with different formulations. Data are presented as mean ± SD (*n* = 6 biological replicates). $^{*}P < 0.05$, $^{**}P < 0.01$, $^{***}P < 0.001$.

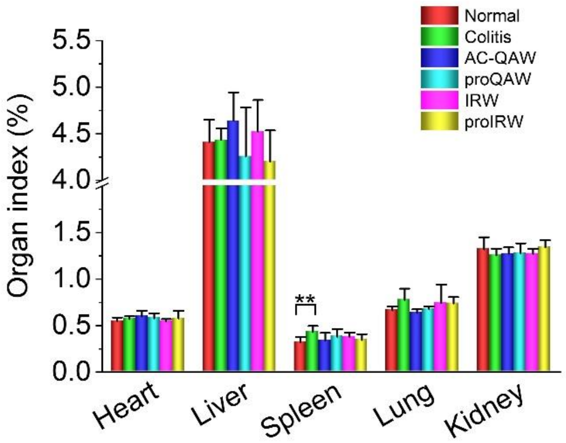

Fig. S30. The organ index values of major organs isolated from mice subjected to different treatments. Data are expressed as mean ± SD (*n* = 6 biological replicates).

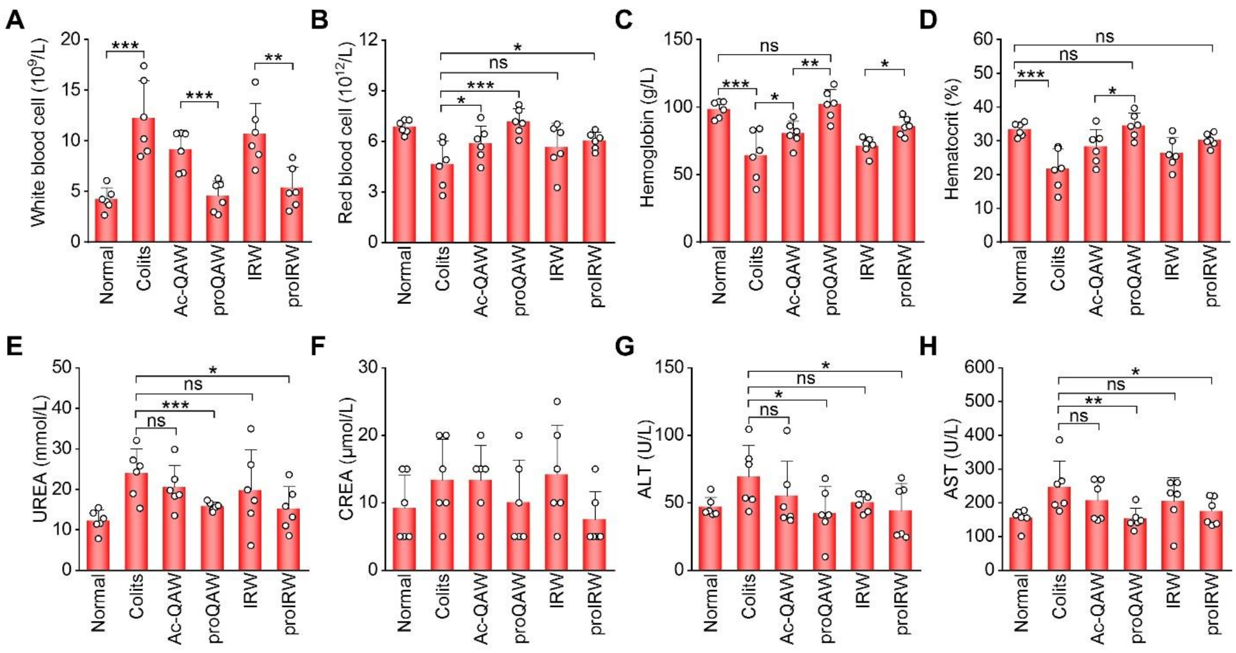

Fig. S31. Quantification of typical hematological parameters and biochemical markers relevant to hepatic and kidney functions of mice following different treatments. (A-D) Levels of white blood cells (A), red blood cells (B), hemoglobin (C), and hematocrit (D). (E-H) The levels of UREA (E), CREA (F), ALT (G), and AST (H). Data are expressed as mean ± SD (n = 6 biological replicates). $^{*}P < 0.05$, $^{**}P < 0.01$, $^{***}P < 0.001$; ns, no significance.

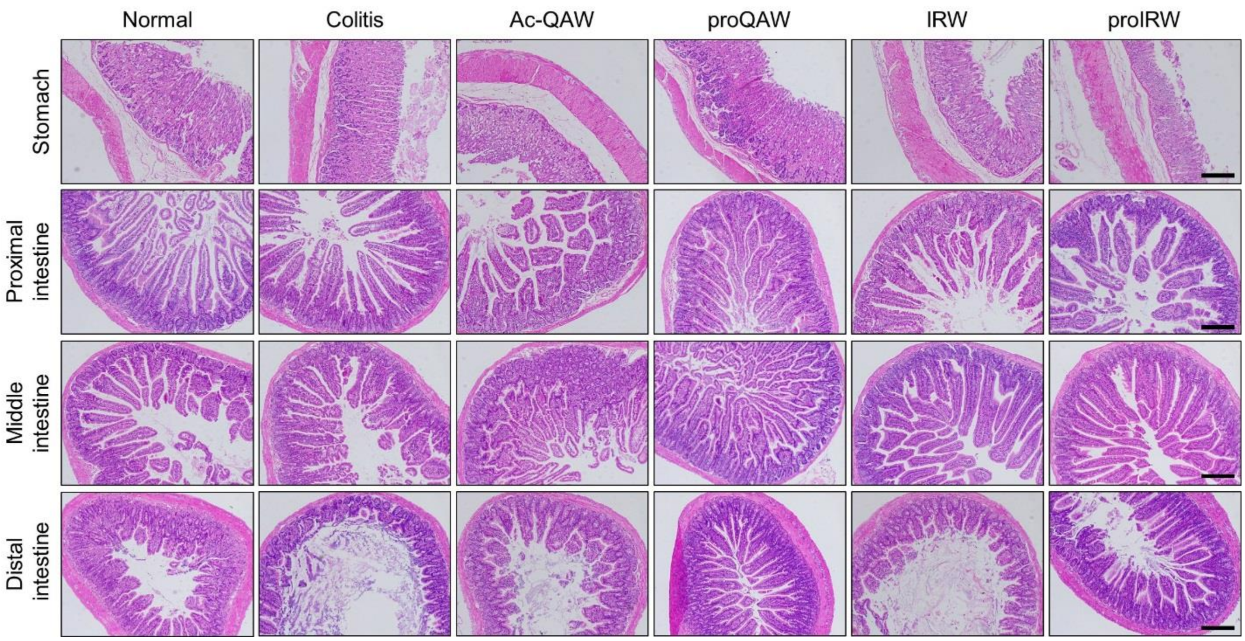

Fig. S32. H&E-stained histological sections of GI tissues from mice with DSS-induced acute colitis after treatment with different formulations. Scale bars, 100 μm.

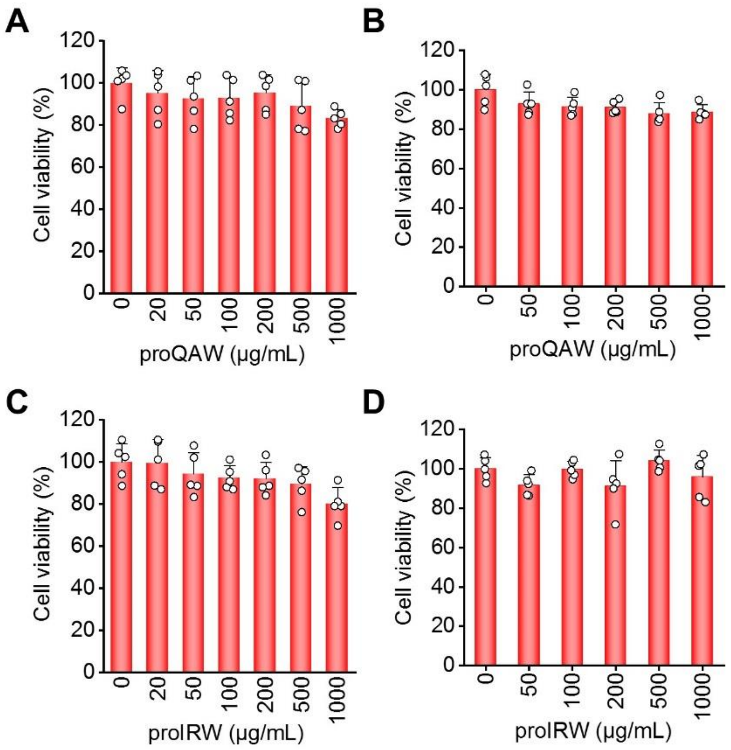

Fig. S33. Cytotoxicity of proQAW and proIRW in different cell lines. (A-B) Cell viability of RAW264.7 macrophages (A) and Caco-2 cells (B) after treatment with different doses of proQAW. (C-D) Cell viability of RAW264.7 macrophages (C) and Caco-2 cells (D) after treatment with different doses of proIRW. After 24 h of incubation with various doses of proQAW or proIRW, cell viability was quantified by CCK8. Data are presented as mean ± SD (*n* = 5).
